# Efficacy and safety of commercial Chinese polyherbal preparations combined with biomedicine for elderly patients with primary insomnia: a systematic review and meta-analysis of randomized controlled trials

**DOI:** 10.3389/fphar.2026.1841014

**Published:** 2026-10-23

**Authors:** Yang Wu, Fang Liu, Mengqi Shi, Lan Yu, Ying Zhang, Huijing Cui, Ruonan Zhang, Peng Xu, Hongmei Nan

**Affiliations:** 1 Department of Traditional Chinese Medicine, School of Changchun University of Chinese Medicine, Changchun, Jilin, China; 2 Department of Graduate Student Administration Office, Affiliated Hospital of Changchun University of Chinese Medicine, Changchun, Jilin, China; 3 Department of Neurology, Affiliated Hospital of Changchun University of Chinese Medicine, Changchun, Jilin, China; 4 Department of Rehabilitation, Nanguan District Hospital of Traditional Chinese Medicine, Changchun, Jilin, China

**Keywords:** combined therapy, commercial Chinese polyherbal preparations, elderly patients, meta-analysis, primary insomnia, traditional Chinese medicine

## Abstract

**Introduction:**

The prevalence of primary insomnia among the elderly is steadily increasing; however, robust evidence supporting the efficacy and safety of combining commercial Chinese polyherbal preparations (CCPPs) with biomedicine therapy remains insufficient. This study aimed to systematically evaluate the efficacy and safety of CCPPs combined with biomedicine versus biomedicine therapy for primary insomnia in elderly patients, and to assess the certainty of the evidence via the GRADE framework and P-curve analysis.

**Methods:**

Seven biomedical databases were searched (before November 2025) for randomized controlled trials (RCTs) involving elderly patients with primary insomnia. Risk of bias was assessed using Cochrane RoB 2 by two researchers independently. Meta-analysis was performed using R software. Outcomes included Pittsburgh Sleep Quality Index (PSQI), total clinical effectiveness rate, Sleep Dysfunction Rating Scale (SDRS), and adverse events. Robustness was evaluated via sensitivity, subgroup, and P-curve analyses.

**Results:**

Meta-analysis suggested that combined therapy was associated with a lower PSQI score (MD = −2.09, 95% CI -2.62 to −1.57) and higher total clinical effectiveness rate (RR = 1.21, 95% CI 1.15–1.27) relative to monotherapy. The combination group also showed a lower risk of adverse events (RR = 0.47, 95% CI 0.36–0.61). However, P-curve analysis revealed notable discrepancies across outcomes: PSQI exhibited sufficient evidential value (Power = 99%), whereas the total clinical effectiveness rate lacked adequate evidential value (Power = 9%), implying potential publication bias. Subgroup analyses showed that heterogeneity decreased within the subgroup of patients with disease duration <1 year, although the between-subgroup difference did not reach statistical significance. The certainty of evidence was rated as “Moderate” for adverse events but “Low” to “Very Low” for efficacy outcomes.

**Conclusion:**

In elderly patients with primary insomnia, combined treatment of CCPPs and biomedicine may help improve sleep quality and may reduce adverse events reported in clinical trials. However, due to methodological limitations, high heterogeneity and potential publication bias among the included trials, the certainty of the available evidence remains limited. Given insufficient evidential value, the reliability of the total clinical effectiveness rate is questionable, and clinicians should place greater emphasis on outcomes assessed using validated scales such as the Pittsburgh Sleep Quality Index (PSQI). Further large-scale, multicenter, double-blind, placebo-controlled randomized controlled trials (RCTs) are needed to further verify these preliminary findings.

**Systematic Review Registration:**

https://www.crd.york.ac.uk/PROSPERO/view/CRD420251186213, identifier: CRD420251186213.

## Graphical Abstract

**Figure:**
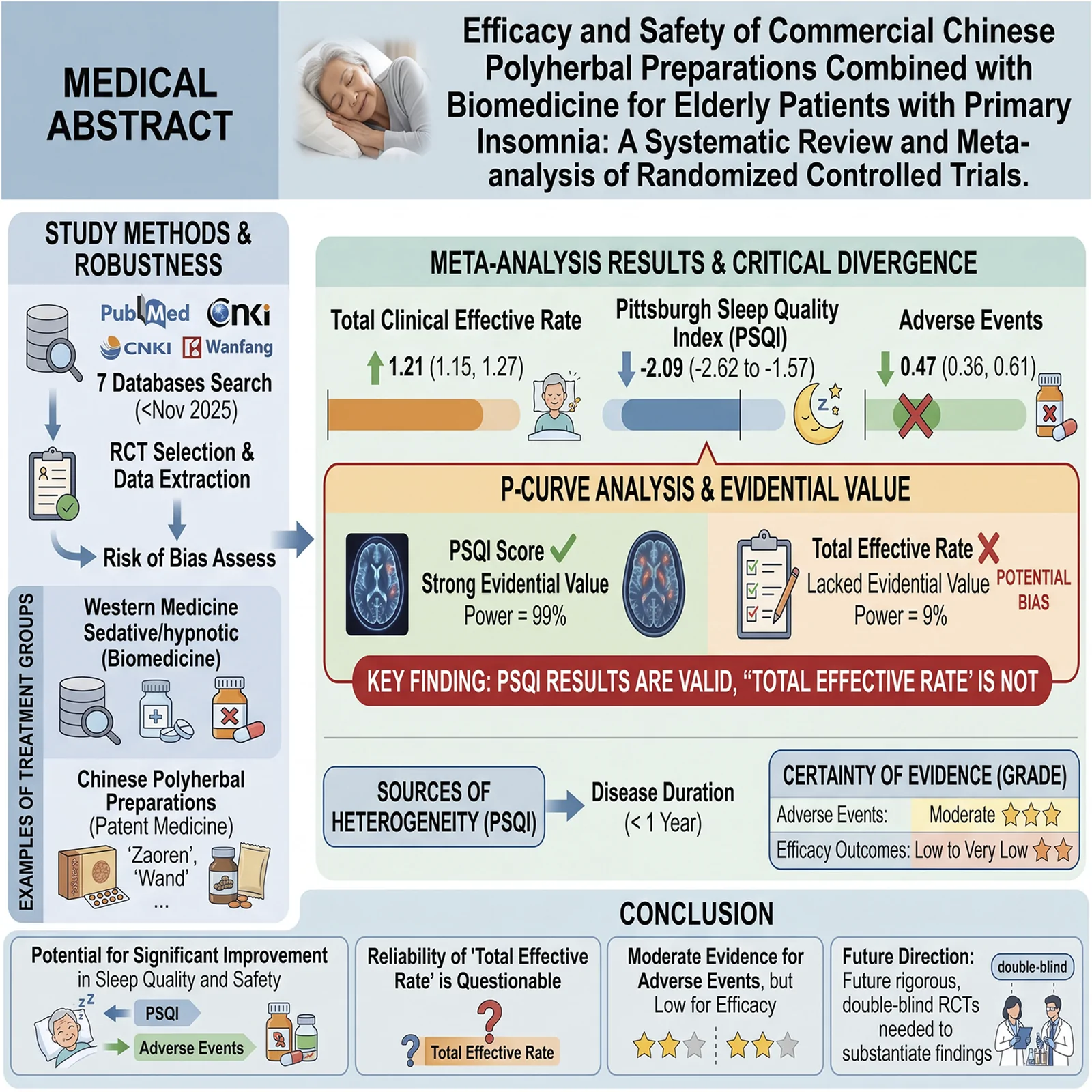

## 1 Introduction

Insomnia disorder is a pervasive and debilitating condition that disproportionately afflicts the aging population. Notably, the classification distinguishing primary insomnia from secondary insomnia originated from early consensus work. As outlined in the ICSD-2, at the 1972 annual meeting of the Association for the Psychophysiological Study of Sleep (APSS), a working group made the first formal attempt to formulate definitions for primary and secondary sleep disorders. Thereafter, insomnia was conventionally divided into primary insomnia and secondary insomnia according to etiology. However, this classification scheme was abandoned in the DSM-5, which integrates both categories into a single diagnostic entity termed insomnia disorder. In this meta-analysis (Cheng et al., 2025), we retained the term primary insomnia because most included RCTs adopted this expression to describe insomnia without secondary causes induced by other physical or mental illnesses, which corresponds to the core population of insomnia disorder defined by the DSM-5. In China, where demographic shifts have resulted in over 190 million individuals aged 65 and above, the prevalence of sleep disturbances in this cohort is staggering, ranging from 40% to 70% (Gu et al., 2025; Song et al., 2025). The clinical sequelae of untreated geriatric insomnia are profound: beyond chronic fatigue and functional impairment, persistent sleep fragmentation significantly precipitates psychiatric morbidity, notably depression, and exacerbates systemic pathologies including cardiovascular disease and cognitive decline (Patel et al., 2018). Studies have shown that secretions produced by chronic sleep disorders may accelerate the progression of Parkinson’s disease (Liu et al., 2025). Consequently, effective management of insomnia is not merely a quality-of-life issue but a critical component of preventative geriatric care. Despite clear clinical guidelines recommending pharmacological interventions-such as benzodiazepines (BZDs) and non-benzodiazepines (NBZDs)-the management of insomnia in elderly patients remains fraught with challenges. The altered pharmacokinetics and physiological vulnerability of older adults significantly narrow the therapeutic window for these agents. Long-term use is increasingly scrutinized due to an unfavorable safety profile characterized by risks of dependency, cognitive blunting, falls, and rebound insomnia (Xu et al., 2024). This dichotomy between the need for symptom relief and the imperative to minimize iatrogenic harm has created an urgent demand for alternative or adjunctive therapeutic strategies. Western hypnotic agents including benzodiazepines and non-benzodiazepines exert sedative effects mainly by targeting the GABA-ergic neurotransmitter system, with a primary therapeutic focus on rapid symptomatic relief of insomnia. In contrast, guided by the TCM principle of syndrome-differentiated treatment, commercial Chinese polyherbal preparations (CCPPs) adopt a holistic therapeutic paradigm rather than aiming merely for sedation. They prioritize the regulation of visceral function, qi-blood and yin-yang to restore the body’s intrinsic homeostatic state. CCPPs should be selected according to patients’ individual TCM syndrome patterns, offering a complementary intervention rationale for the complex pathogenesis of insomnia among older adults.

CCPPs are increasingly applied in integrated regimens for insomnia treatment. Unlike conventional single-target hypnotic agents, some CCPPs exert complex pharmacological actions. Preclinical pharmacological studies have reported that certain CCPPs preparations may modulate neurobiological pathways including the gut-brain axis and tryptophan metabolism, which may contribute to their clinical benefits (Tian et al., 2025; Shu et al., 2026). In clinical practice of Traditional Chinese Medicine, elderly patients with insomnia can be further classified according to their clinical manifestations. Common syndrome patterns include heart-kidney disharmony, liver-qi stagnation, heart-blood stasis, heart-liver blood deficiency, and liver-yang hyperactivity. Given that each herbal medicine possesses its unique nature, flavour and meridian tropism, CCPPs formulated with distinct herbal combinations can deliver targeted and individualized treatment corresponding to these different TCM syndromes. This fully embodies the core TCM therapeutic principle of treatment based on syndrome differentiation and holistic regulation of visceral functions as well as qi-blood. In European phytotherapy, botanical drug preparations derived from *Valeriana officinalis* L. and *Humulus lupulus* L. are well-established interventions for insomnia. In contrast to these mostly single-ingredient European botanical drug products, CCPPs adopt multi-ingredient holistic combinatorial strategies rooted in traditional Chinese medicine theories.

While numerous randomized controlled trials (RCTs) suggest that combining CCPPs with biomedicine may enhance efficacy and reduce adverse events (Zhao et al., 2025; Yue, 2023), the existing evidence base is fragmented. Prior systematic reviews have often conflated primary and secondary insomnia or failed to stratify by age, obscuring the specific benefits for elderly patients with primary insomnia-a distinct clinical entity unconfounded by somatic comorbidities.

To address this uncertainty and inform clinical practice, we conducted a systematic review and meta-analysis to evaluate the efficacy and safety of combining CCPPs with biomedicine versus biomedicine alone in elderly patients with primary insomnia. This study distinguishes itself through rigorous methodological standards: we employed P-curve analysis to test the evidential value and detect potential p-hacking, and utilized the GRADE framework to assess the certainty of evidence. By adhering to the PRISMA 2020 guidelines (Page et al., 2021), we aim to provide a robust, evidence-based synthesis to guide therapeutic decision-making in this vulnerable population.

## 2 Methods

### 2.1 Protocol and registration

This systematic review and meta-analysis was conducted in strict adherence to the *Preferred Reporting Items for Systematic Reviews and Meta-Analyses* (PRISMA) 2020 statement (Page et al., 2021). The study protocol was prospectively registered with PROSPERO (Registration number: CRD420251186213).

### 2.2 Data sources and search strategy

Comprehensive systematic searches were conducted across four Chinese databases (Wanfang, CNKI, VIP, Chinese Biomedical Database [CBM]) and three English databases (PubMed, Embase, Cochrane Library). Complete independent search strategies were developed for each database according to their respective retrieval syntax, and the full search formulas are presented in Supplementary Appendix S1. The search window spanned from the inception of each database to November 2025. To ensure maximum sensitivity, we employed a combination of Medical Subject Headings (MeSH) terms and free-text keywords, including: (“Randomized Controlled Trial” OR “RCT”) AND (“Traditional Chinese Medicine” OR “Chinese Patent Medicine” OR “CPM”) AND (“Insomnia” OR “Primary Insomnia”) AND (“Elderly” OR “Aged”). No language restrictions were imposed, though only English and Chinese studies were retrieved.

### 2.3 Eligibility criteria

Studies were selected based on the following PICOS criteria:

Participants (P): Patients aged ≥60 years with a definitive diagnosis of primary insomnia according to recognized diagnostic guidelines (e.g., DSM-5, ICD-10, or CCMD-3, and others). Patients with insomnia secondary to severe somatic diseases (e.g., cancer, stroke, heart failure) or psychiatric disorders (e.g., schizophrenia) were excluded to minimize confounding.

Interventions (I): The experimental group received CCPPs combined with the same biomedicine regimen used in the control group. CCPPs in this study refer to standardized finished botanical drug products officially approved and marketed by national drug regulatory authorities. They are manufactured in accordance with established TCM prescriptions under standardized production procedures, including capsules, tablets, soft capsules, granules, oral liquids, pills, etc. Detailed taxonomic validation information for each included CCPP, including full validated species binomial with author citation, family assignment and corresponding pharmacopoeial botanical drug name, is provided in Supplementary Appendix S2 of the main manuscript. This study explicitly excludes hospital-prepared TCM preparations, self-formulated botanical drug decoctions, and non-pharmacological TCM interventions such as acupuncture and tuina. All interventions labelled as CCPPs in the included RCTs were double-checked against official drug-approval databases to confirm they were officially marketed proprietary Chinese medicines.

Comparators (C): The control group received biomedicine monotherapy. Control interventions consisted of conventional biomedical hypnotics, primarily benzodiazepines (e.g., estazolam, alprazolam) and non-benzodiazepines (e.g., zolpidem, zopiclone), which are first-line clinical agents for geriatric insomnia.

Outcomes (O):

*Primary Outcomes:* Sleep quality assessed by the Pittsburgh Sleep Quality Index (PSQI).

*Secondary Outcomes:* Total clinical effectiveness rate (defined by validated reduction in symptom scores), Adverse events (AEs) and Sleep Dysfunction Rating Scale (SDRS) scores.

Study Design (S): Only Randomized Controlled Trials (RCTs) were included.

### 2.4 Exclusion criteria

Studies were excluded if they met any of the following conditions: (1) Non-RCTs, including reviews, case reports, animal studies, or conference abstracts; (2) Interventions involving non-pharmacological TCM therapies (e.g., acupuncture, tuina), non-marketed traditional Chinese medicinal preparations (non-CCPPs), or studies with unclear medication details; (3) Duplicate publications or studies with insufficient data for extraction.

### 2.5 Data extraction and management

Two independent reviewers (Y.W. and F.L.) screened titles and abstracts, followed by full-text review. Data were extracted using a standardized form in EndNote 25, capturing: first author, publication year, sample size, age/gender distribution, diagnostic criteria, baseline PSQI, CCPPs dosage, western medication dosage, TCM syndrome type, formula-function category, follow-up duration, intervention details (dosage, duration), and outcome measures. Any discrepancies were resolved through discussion or consultation with a third senior reviewer.

### 2.6 Risk of bias assessment

The methodological quality of the included RCTs was assessed using the Cochrane Risk of Bias 2 (RoB 2) tool. Five domains were evaluated: (1) randomization process; (2) deviations from intended interventions; (3) missing outcome data; (4) measurement of the outcome; and (5) selection of the reported result. Each study was classified as having “low risk,” “some concerns,” or “high risk” of bias.

### 2.7 Statistical analysis

All statistical analyses were performed using R software (Version 4.5.2, The R Foundation).

Effect Measures: Dichotomous outcomes (e.g., total clinical effectiveness rate, adverse events) were expressed as Risk Ratios (RR) with 95% Confidence Intervals (CI). Continuous outcomes (e.g., PSQI scores, SDRS) were expressed as Mean Difference (MD) or Standardized Mean Difference (SMD) with 95% CIs. SMD was selected when different scales were used to measure the same outcome.

Heterogeneity: Heterogeneity was assessed using the Cochran Q test and the I^2^ statistic. A fixed-effects model was applied if heterogeneity was low (P ≥ 0.10, I^2^ ≤ 50%); otherwise, a random-effects model was utilized.

Subgroup and Sensitivity Analysis: To explore the sources of heterogeneity, pre-specified subgroup analyses were performed according to treatment duration (≤8 weeks vs. > 8 weeks), disease duration (<1 year vs. 1–3 years), and biomedicine class. In addition, we planned to conduct exploratory subgroup analyses stratified by TCM syndrome pattern and formula-function category of CCPPs for the PSQI outcome. However, since most included RCTs did not explicitly report TCM syndrome differentiation of participants, only the subgroup analysis based on formula-function category was ultimately carried out. Sensitivity analysis was performed using the “leave-one-out” method to test the robustness of the pooled effect estimates.

Publication Bias and Evidential Value: Publication bias was evaluated using funnel plots and Egger’s regression test. The “trim-and-fill” method was used to adjust for potential bias. Additionally, P-curve analysis was conducted to assess whether the distribution of statistically significant p-values indicated true evidential value or p-hacking. For P-curve computation, only statistically significant p-values derived from the two predefined primary outcomes (PSQI and total clinical effectiveness rate) were included. Multiple secondary outcomes and non-independent replicated results originating from the same trial were strictly excluded to avoid analytical bias.

### 2.8 Certainty of evidence assessment

The certainty of the evidence for each outcome was appraised using the GRADE (Grading of Recommendations Assessment, Development, and Evaluation) approach. Evidence was graded as “high,” “moderate,” “low,” or “very low” based on five domains: risk of bias, inconsistency, indirectness, imprecision, and publication bias.

## 3 Results

### 3.1 Study selection and baseline characteristics

The systematic search identified 4,392 initial records. Following deduplication and strict eligibility screening, 16 randomized controlled trials (RCTs) involving 1,353 participants were included in the final analysis (Figure 1). All studies were conducted in China and published between 2011 and 2025.

**FIGURE 1:**
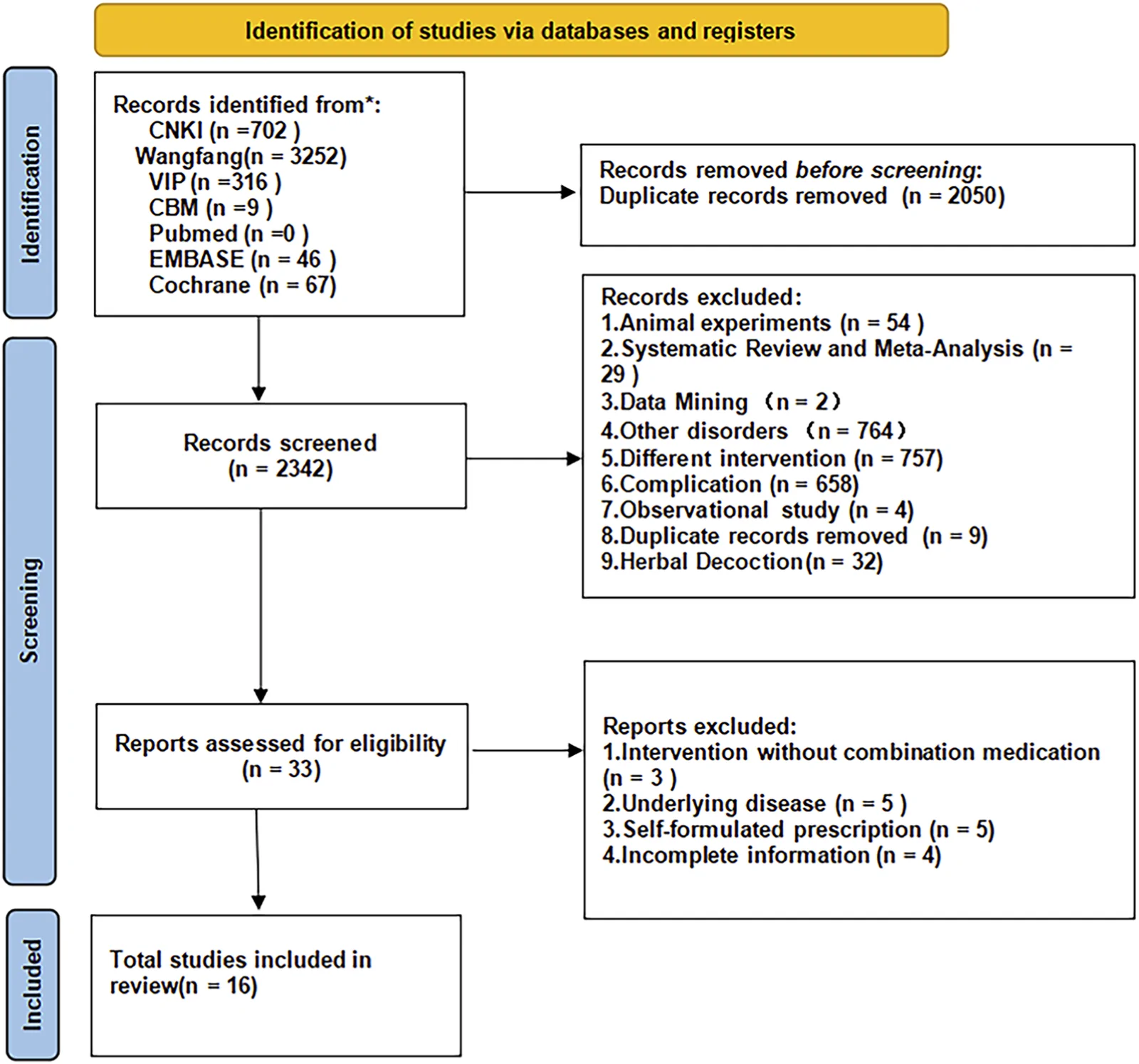
Flow chart of study selection.

The study population comprised elderly patients aged 60–89 years diagnosed with primary insomnia. Participants were randomized to receive either Commercial Chinese Polyherbal Preparations (CCPPs) combined with biomedicine (*n* = 678) or biomedicine monotherapy (*n* = 675). The duration of intervention ranged from 30 to 90 days. Detailed characteristics of the included trials are presented in Table 1.

**TABLE 1:** Basic characteristics of the included studies.

Source	Year	Sample		Sex (M/F)		Age (Years, x̅ ± s)		Diagnostic criteria	Disease duration (Years/months, x̅ ± s)	
		T	C	T	C	T	C		T	C
Wang & Wang	2014	45	45	24/21	25/20	68.3 ± 2.1	69.2 ± 2.9	AASM; PSQI>7	3.0 ± 0.6years	2.9 ± 1.0years
Xiong & Cai	2015	42	42	20/22	18/24	70.7 ± 1.8	70.3 ± 1.5	Not mentioned	Not mentioned	Not mentioned
Yu LJ	2023	44	43	25/19	24/19	74.24 ± 2.98	74.15 ± 2.91	CGDT-AI (2017)	15.59 ± 1.85 months s	15.52 ± 1.79 months
Xu & Zhou	2022	48	48	34/14	31/17	71.01 ± 6.73	71.36 ± 6.98	CGAI-2012	3.50 ± 0.43months	3.72 ± 0.40months
Wang NN et al.	2014	80	80	41/39	38/42	69 ± 12	68 ± 12	CCMD-3	Total score: NR	Total score: NR
Cheng XJ et al.	2025	24	24	9/15	11/13	72.38 ± 5.04	73.46 ± 5.23	CGDTI; WHO/WPO	14.46 ± 5.98months	13.68 ± 5.88months
Xie GJ et al.	2017	35	35	14/21	16/19	71.2 ± 11.0	71.3 ± 10.2	2012-CGDT-AI	Not mentioned	Not mentioned
Huang YL	2023	30	30	16/14	18/12	89.26 ± 0.32	89.15 ± 0.28	Integrated TCM-WM clinical guidelines for insomnia disorder	5.49 ± 1.88 years	5.70 ± 1.79years
Yang ZY	2017	30	30	7/23	7/23	70.2 ± 6.4	70.9 ± 6.9	CCMD-3; TCM criteria (Bu-Mie)	≥1 year	≥1year
Zheng CJ	2024	36	36	22/14	23/12	70.23 ± 3.27	70.54 ± 4.32	CGAI-2017	4.65 ± 1.08 months	4.83 ± 1.24 months
Zhao & Qiu	2015	56	56	22/34	26/30	68.9 ± 5.1	69.7 ± 4.1	CCMD-3	21.8 ± 5.2 months	24.1 ± 4.3months
Peng YY	2013	50	50	30/20	24/26	73.5 ± 26.6	73.2 ± 24.5	Not mentioned	7.65 ± 2.67 years	7.13 ± 2.01year
Zhao LJ et al.	2011	42	40	19/23	18/22	70.52 ± 5.29	71.63 ± 6.08	CCMD-3	16.83 ± 7.38months	15.60 ± 6.19months
Ding YQ	2016	41	41	20/21	22/19	72.6 ± 27.8	73.88 ± 24.56	ICD-10	7.28 ± 2.61year	7.36 ± 2.78years
Peng & Tu	2021	35	35	15/20	16/19	69.41 ± 3.09	69.35 ± 3.17	2017 Chinese adult insomnia guideline	16.45 ± 2.41month	16.31 ± 2.36months
Wang T et al.	2014	40	40	17/20	15/19	63.3 ± 9.8	63.1 ± 8.6	CCMD-3	31.9 ± 16.2months	31.7 ± 14.5months

Baseline PSQI (x̅ ± s)		Intervention		CCPPs dosage	Western medication dosage	Formula-function category	Intervention period	TCM syndrome type	Follow-up duration	Outcome
T	C	T	C							
14.13 ± 0.60	14.02 ± 0.61	C + Shensong Yangxin capsule	Zopiclone	SYC 4 capsules tid po	Zo 3 mg/d - 6 mg/d within 1w po	Replenish qi-nourish heart-calm mind	12-week	Not mentioned	No post-treatment follow-up	①②③⑤⑥
Not mentioned	Not mentioned	C + Qiye Anshen Tablet (+Music)	Zopiclone (+Music)	QYA 1 tablet tid po	Zo 7.5 mg qd po	Nourish blood-calm mind	4-week	Not mentioned	No post-treatment follow-up	①③⑦
Total score: NR	Total score: NR	C + Zaoren Anshen oral liquid	Estazolam	ZAS-liquid 20 mL qd po	Est 2 mg qd po	Nourish blood-calm mind	4-week	Heart-liver blood deficiency type insomnia	No post-treatment follow-up	①②③④
14.89 ± 3.15	14.07 ± 3.34	C + Zaoren Anshen granules	Eszopiclone	ZAS-Gran 1 sachet qd po	Esz 2 mg qd po	Nourish blood-calm mind	4-week	Not mentioned	No post-treatment follow-up	①②③
Not mentioned	Not mentioned	C + Yindan Xinnaotong soft capsule	Zopiclone	YDX 4 mg qd po	Zo 3 mg qd po	Promote blood circulation	4-week	Not mentioned	No post-treatment follow-up	①③⑦
17.29 ± 1.70	17.17 ± 1.68	C + Yishen Yangxin Anshen Tablet	Estazolam; Clonazepam; Zolpidem; alprazolam	YSYX 4 tablets tid po	Zo + Est + Cl + Al 1 tablets qn po	Replenish heart-kidney, calm mind	4-week	Heart-kidney non-interaction	No post-treatment follow-up	①②④
6.45 ± 0.88	6.25 ± 0.84	C + Tianwang Buxin Pellet	Eszopiclone	TWBXD 8 g bid po	Esz 2 mg qn po	Nourish yin-replenish heart-calm mind	4-week	Yin-deficiency and blood-insufficiency-insomnia	No post-treatment follow-up	①②
Total score: NR	Total score: NR	C + Wuling capsule	Estazolam	Wl capsule 0.99 g tid po	Est 2 mg qn po	Tonify kidney-nourish heart-calm mind	1-month	Not mentioned	No post-treatment follow-up	①②
11.33 ± 2.94	11.50 ± 4.34	C + Songling Xuemaikang capsule	Estazolam; Alprazolam; Clonazepam	SLXMK capsule 1.5 g tid po	Est 1 mg + Al 0.4 mg + Cl 2 mg	Pacify liver-suppress yang	90-day	Not mentioned	No post-treatment follow-up	②
14.13 ± 3.16	14.32 ± 2.98	C + Shumian capsule	Eszopiclone	SM capsule 3 capsules bid po	Esz 2 mg qd po	Soothe liver-relieve stagnation-calm mind	4-week	Not mentioned	No post-treatment follow-up	①②③
17.14 ± 1.32	16.89 ± 1.45	C + Shumian capsule	Mirtazapine	SM capsule 3 capsules bid po	Mi 30 mg qn po	Soothe liver-relieve stagnation-calm mind	4-week	Not mentioned	No post-treatment follow-up	①②③
Not mentioned	Not mentioned	C + Shumian capsule	Oxazepam	SM capsule 3 capsules pre-dinner +3 capsules at bedtime	Ox 30 mg po	Soothe liver-relieve stagnation-calm mind	4-week	Not mentioned	No post-treatment follow-up	①
15.88 ± 2.85	15.65 ± 2.82	C + Qiye Shen’an Tablet	Zopiclone	QYAS 50 mg tid po	ZO 3.75 mg qn po	Nourish blood-calm mind	4-week	Not mentioned	No post-treatment follow-up	①②③
Total score: NR	Total score: NR	C + Bailemian capsule	Oxazepam	BLM 4 tablets bid po	Ox 30 mg qd po	Nourish yin-soothe liver-calm mind	4-week	Not mentioned	No post-treatment follow-up	①②③
Not mentioned	Not mentioned	C + Bailemian capsule	Alprazolam	BLM 4 capsules bid po	Al 0.8 mg qn po	Nourish yin-soothe liver-calm mind	4-week	Not mentioned	No post-treatment follow-up	①③⑦
Not mentioned	Not mentioned	C + Yimianda Tablet	Zopiclone	YMD 5 tablets bid po	Zo 3.75 mg qn po	Nourishing yin and nourishing blood	4-week	Not mentioned	No post-treatment follow-up	①②③

T = Treatment group C=Control group NA = Not applicable ①Response rate ②Pittsburgh Sleep Quality Index (PSQI)③Adverse reactions ④Traditional Chinese Medicine (TCM) syndrome evaluation criteria ⑤SAS ⑥SDS ⑦SDRS.

AASM: american academy of sleep medicine; SYC: shensong yangxin capsule; Zo: Zopiclone; CGDT-AI (2017): Chinese Guidelines for the Diagnosis and Treatment of Adult Insomnia (2017 Edition); ZAS-liquid: Zaoren Anshen Liquid Est:Estazolam; Esz: Eszopiclone; CCMD-3: Chinese Classification and Diagnostic Criteria of Mental Disorders-3; YDX: yindan xinnaotong soft capsule; Zo: Zopiclone; CGDTI: Chinese Guidelines for Diagnosis and Treatment of Insomnia; WHO/WPO: WHO/WPO, Clinical Practice Guideline for TCM, of Insomnia; YSYX: yishen yangxin anshen tablet; Zo: Zolpidem; Cl: Clonazepam; Al: Alprazolam; 2012-CGDT-AI: 2012 Chinese Guidelines for Diagnosis and Treatment of Adult Insomnia; TWBXD: tianwang buxin pellet; Wl capsule: Wuling capsule; CGAI-2012: Chinese guidelines for the diagnosis and treatment of adult insomnia; CCMD-3: Chinese classification and diagnostic criteria of mental disorders, Third Edition (CCMD-3); TCM, Criteria (Bu-Mie): Diagnostic and Therapeutic Effect Criteria for TCM, Diseases and Syndromes (Bu-Mie); Mi: Mirtazapine; SM, Capsule: Shumian Capsule; Ox: Oxazepam; QYAS: Qiye Shen’an Tablet; ICD-10: International Statistical Classification of Diseases and Related Health Problems, 10th Revision; BLM: bailemian capsule; Ox: Oxazepam; YMD: yimianda tablet.

### 3.2 Methodological quality assessment

The risk of bias assessment for the 16 included studies was conducted using the Cochrane Risk of Bias tool (RoB2), strictly following each evaluation criterion. The results are as follows: 1) Randomization process: All included studies adopted random allocation, yet eight studies (Ding, 2016; Peng, 2013; Wang N. N. et al., 2014; Xie et al., 2017; Yu, 2023; Zhao and Qiu, 2015; Zhao et al., 2011; Wang N. N. et al., 2014) did not specify the specific method of randomization, leading to a some concerns rating. The remaining studies were rated as low risk. 2) Deviations from the intended interventions: None of the included trials reported blinding of participants or researchers. As several primary outcomes relied on subjective symptom assessment, the absence of blinding created substantial risk of performance bias. Accordingly, all studies were rated at high risk for this domain. 3) Missing outcome data: Outcome data were complete in all trials, with dropout rates within acceptable limits. All studies were classified as low risk. 4) Measurement of the outcome: All outcome indicators were subjective, so all trials were rated at high risk. 5) Selection of the reported result: Most original trials lacked prospective trial registration. Five studies (Ding, 2016; Huang, 2023; Peng, 2013; Yu, 2023; Wang T. et al., 2014) provided insufficient evidence to verify prespecified outcome reporting and were rated high risk. All other trials were judged to raise some concerns. The overall risk of bias for each individual study was assessed as high risk (Figure 2; Figure 3).

**FIGURE 2:**
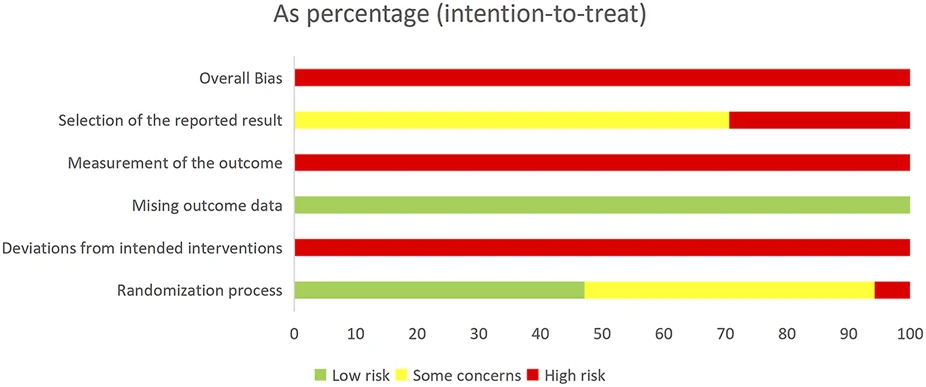
Risk-of-bias bar chart.

**FIGURE 3:**
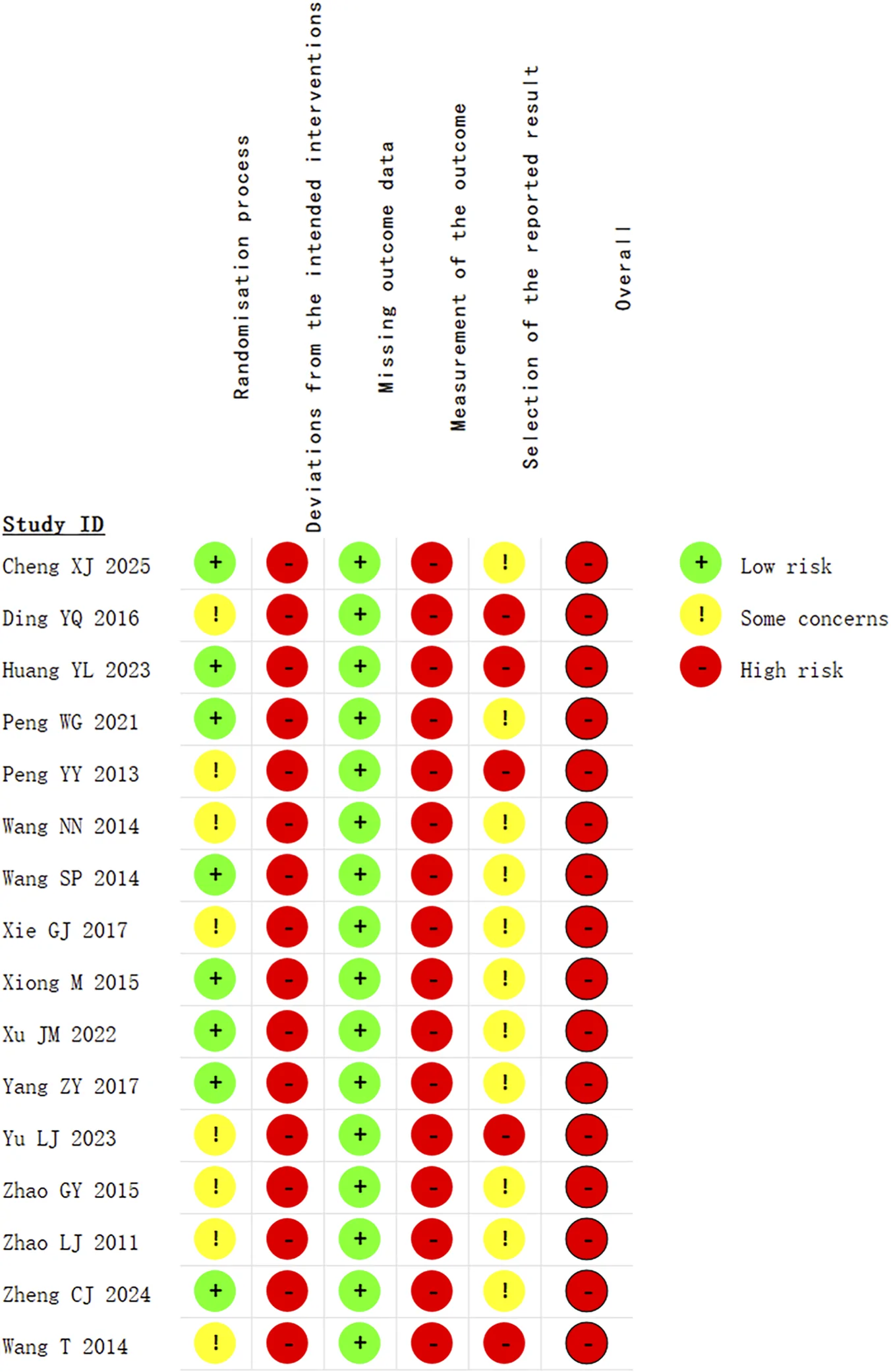
Risk-of-bias summary plot.

### 3.3 Primary efficacy outcomes

#### 3.3.1 Pittsburgh Sleep Quality Index (PSQI)

Eight trials (n = 630) reported valid PSQI scores. Substantial heterogeneity was detected across studies (I^2^ = 90.0%, P < 0.0001); therefore, a random-effects model was adopted. Since all eight trials employed the standard 0–21 point PSQI scale, the mean difference (MD) was selected as the effect measure to improve clinical interpretability. Random-effects meta-analysis demonstrated that combination therapy yielded a significant reduction in PSQI scores (MD = −2.09, 95% CI −2.62 to −1.57; Figure 4). Given the extreme between-study heterogeneity, this pooled estimate should be interpreted with considerable caution.

**FIGURE 4:**
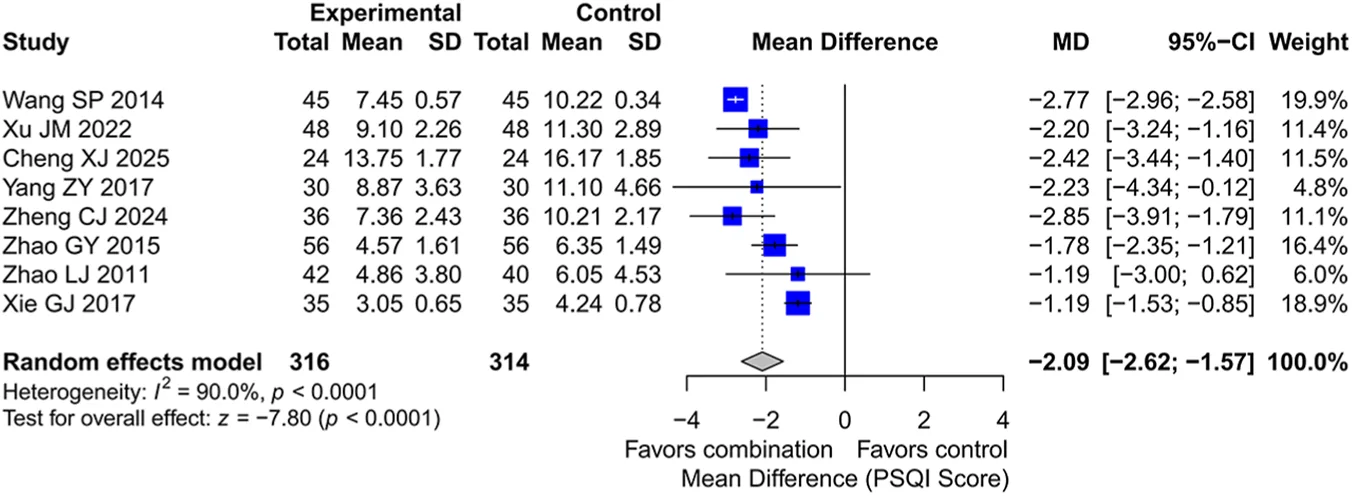
Forest plot of pooled mean difference for Pittsburgh Sleep Quality Index (PSQI) scores.

### 3.4 Secondary outcomes

#### 3.4.1 Total clinical effectiveness rate

Data from 15 studies (n = 1,284) were pooled to assess the total clinical effectiveness rate. Heterogeneity among studies was low (*I* ^2^ = 12.0%, P = 0.32). The fixed-effects model indicated that combination therapy was associated with a statistically significant increase in the clinical response rate compared with monotherapy (Risk Ratio [RR] 1.21, 95% CI 1.15 to 1.27; Figure 5).

**FIGURE 5:**
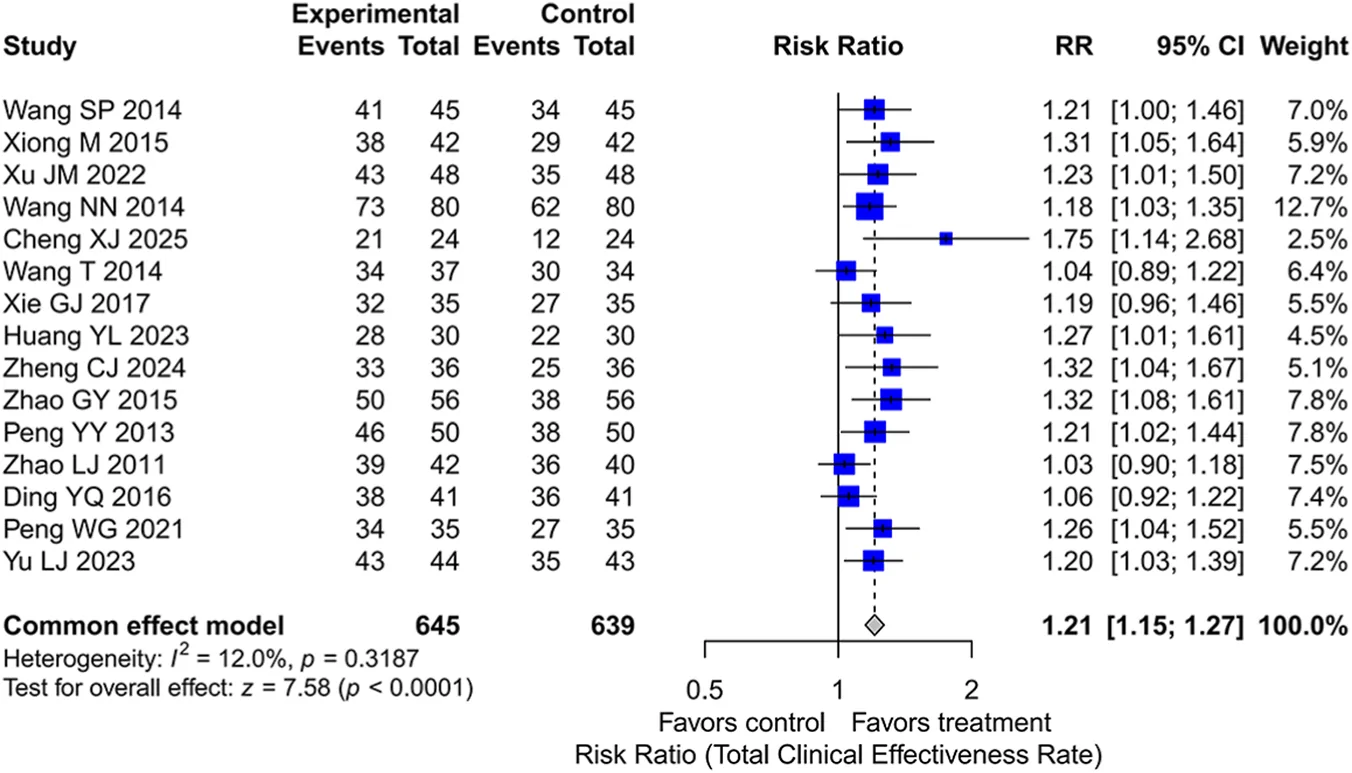
Forest plot of pooled risk ratio for total clinical effectiveness rate.

Analysis of efficacy grades revealed a favorable shift in the intervention group: the proportion of patients achieving “Cured” or “Markedly Effective” status was significantly higher in the combination group (64.7%) compared to the control group (47.6%), while the rate of treatment failure (“Ineffective”) was reduced from 23.8% to 7.8% (Supplementary Figure S1; Supplementary Figure S2).

#### 3.4.2 Safety profile (adverse events)

Adverse events (AEs) were reported in 11 studies (n = 1,006). Heterogeneity was negligible (*I* ^2^ = 0.4%). The incidence of AEs was 11.3% (57/506) in the combination group versus 24.4% (122/500) in the control group. Combined therapy was significantly associated with a lower risk of documented adverse events (RR = 0.47, 95% CI 0.36 to 0.61, P < 0.001; Figure 6). The most frequently reported AEs included mild nausea, dizziness, and somnolence, all of which were transient. Of note, none of the included RCTs prospectively assessed geriatric-specific high-risk adverse events, such as falls, cognitive impairment, excessive sedation, drug dependence, and withdrawal reactions. Detailed characteristics of adverse events are presented in Table 2. However, this finding should be interpreted with caution owing to confounding factors such as selective reporting of adverse events, inadequate safety monitoring, and potential differences in hypnotic dosage between groups. Notably, no included trials investigated potential drug-drug interactions between CCPPs-derived botanical metabolites and biomedical hypnotic agents. Furthermore, systematic monitoring of hepatic and renal function and other organ-related adverse events was not performed in any primary study, which limits the comprehensive safety evaluation of combination therapy in elderly patients.

**FIGURE 6:**
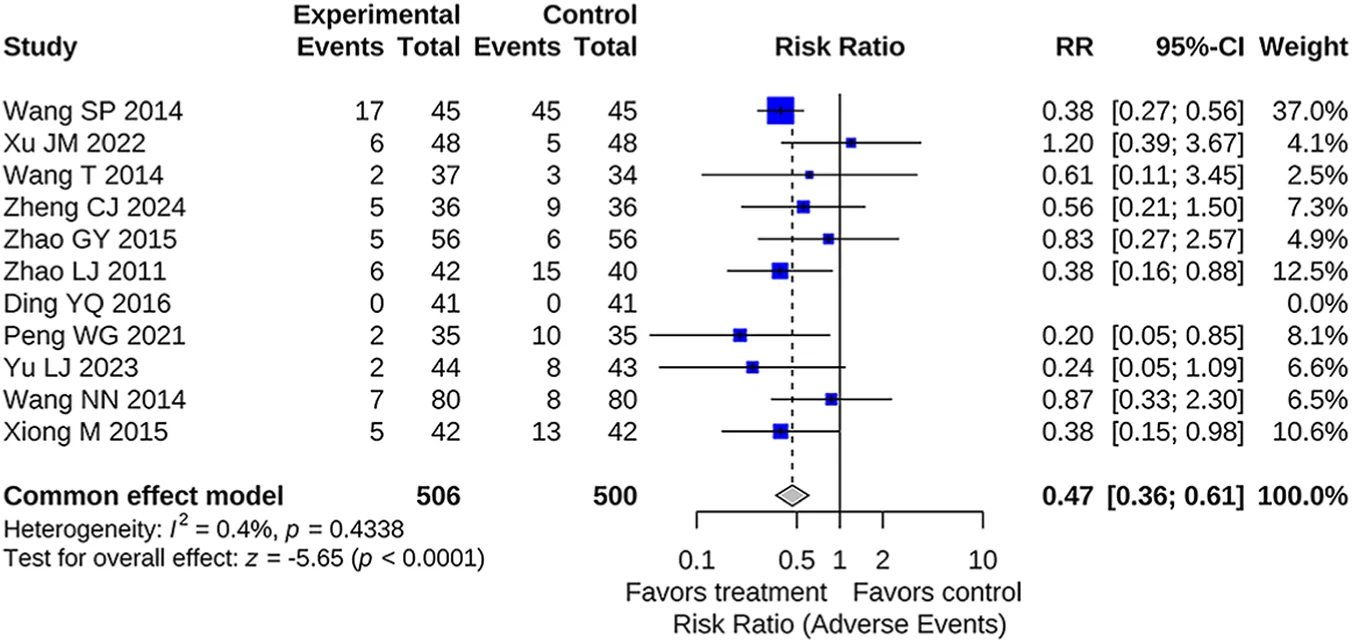
Forest plot of pooled risk ratio for adverse events.

**TABLE 2:** Basic characteristics of adverse events.

Name (year)	N (Exp/Con)	Severe fatigue	Somnolence	Dizziness and headache	Gastrointestinal adverse reactions	Constipation	Dry mouth and bitter taste	Decreased appetite	Muscular discomfort
Wang and Wang, 2014	45/45	3/7	2/8	2/9	5/8	3/5	2/8	NR	NR
Xiong and Cai, 2015	42/42	0/2	NR	NR	2/1	NR	1/2	NR	2/3
Yu 2023	44/43	1/2	1/2	0/2	NR	NR	0/2	NR	NR
Xu and Zhou, 2022	48/48	1/2	NR	3/2	2/1	NR	NR	NR	NR
Wang N. N. et al., 2014	80/80	NR	1/1	2/3	1/2	NR	3/2	NR	NR
Zheng, 2024	36/36	NR	1/2	2/3	2/4	NR	NR	NR	NR
Zhao and Qiu, 2015	56/56	0/1	3/2	1/1	1/2	NR	NR	NR	NR
Peng and Tu, 2021	35/35	0/3	NR	1/2	1/3	NR	NR	0/2	NR

Exp = combination group (CCPM plus Biomedicine); Con = Biomedicine-only group. NR = not reported. None of the included RCTs prospectively monitored or reported geriatric-specific high-risk adverse outcomes including falls, cognitive impairment, excessive sedation, drug dependence, withdrawal reactions, or liver-renal dysfunction.

#### 3.4.3 Sleep Dysfunction Rating Scale (SDRS)

Three studies (n = 314) assessed sleep dysfunction using the Sleep Dysfunction Rating Scale (SDRS). The pooled analysis showed that combined therapy was associated with a reduction in SDRS scores, although the difference did not reach statistical significance (SMD = −0.98, 95% CI −2.08 to 0.11; I^2^ = 92.7%; Supplementary Figure S3), suggesting a potential benefit to sleep quality.

### 3.5 Investigation of heterogeneity (subgroup analysis)

Given the high heterogeneity in PSQI scores (I^2^ = 90%), prespecified subgroup analyses were performed (Figure 7, Figure 8; Supplementary Figure S4; Supplementary Figure S5). Stratification by disease duration resulted in markedly reduced heterogeneity within the subgroup of patients with disease duration <1 year (I^2^ = 0%), though the pooled effect in this subgroup did not reach statistical significance. High heterogeneity remained in the 1–3 years disease-duration subgroup (I^2^ = 92.7%), and the test for subgroup differences was non-significant (P = 0.1996). Subgroup analysis by treatment duration showed that heterogeneity was eliminated in the >8-week subgroup (I^2^ = 0%), while substantial heterogeneity persisted for trials with treatment duration ≤8 weeks (I^2^ = 67.1%); the difference between subgroups reached statistical significance (P = 0.0012). Subgrouping according to Western drug class failed to fully explain between-study heterogeneity, with a non-significant subgroup difference (P = 0.1088). Beyond the predefined stratifications, an exploratory subgroup analysis by TCM syndrome pattern (classified based on the therapeutic indications of included CCPPs) was conducted for PSQI outcomes. The test for subgroup differences revealed no statistically significant difference in treatment effects across TCM syndrome subgroups (X^2^ = 0.56, df = 4, P = 0.9673). Statistically significant reductions in PSQI scores were observed in the heart-liver blood deficiency subgroup (MD = −2.20, 95%CI −3.24 to −1.16) and the liver yang ascendant generating internal wind subgroup (MD = −2.23, 95%CI −4.34 to −0.12). By contrast, the 95% confidence intervals crossed zero for stasis of heart blood (MD = −2.25, 95%CI −11.69 to 7.19; I^2^ = 65.3%), heart-kidney disharmony (MD = −1.71, 95%CI −9.42 to 6.01), and liver constraint with qi stagnation (MD = −2.22, 95%CI −8.90 to 4.47), indicating non-significant pooled effects in these subgroups. It should be highlighted that most syndrome subgroups contained only one or two trials, which limits statistical power. Moreover, the classification of TCM syndrome patterns relied on CCPPs’ indications rather than patient-specific syndrome differentiation recorded in the original trials; therefore, these findings should be interpreted cautiously as exploratory evidence rather than definitive conclusions.

**FIGURE 7:**
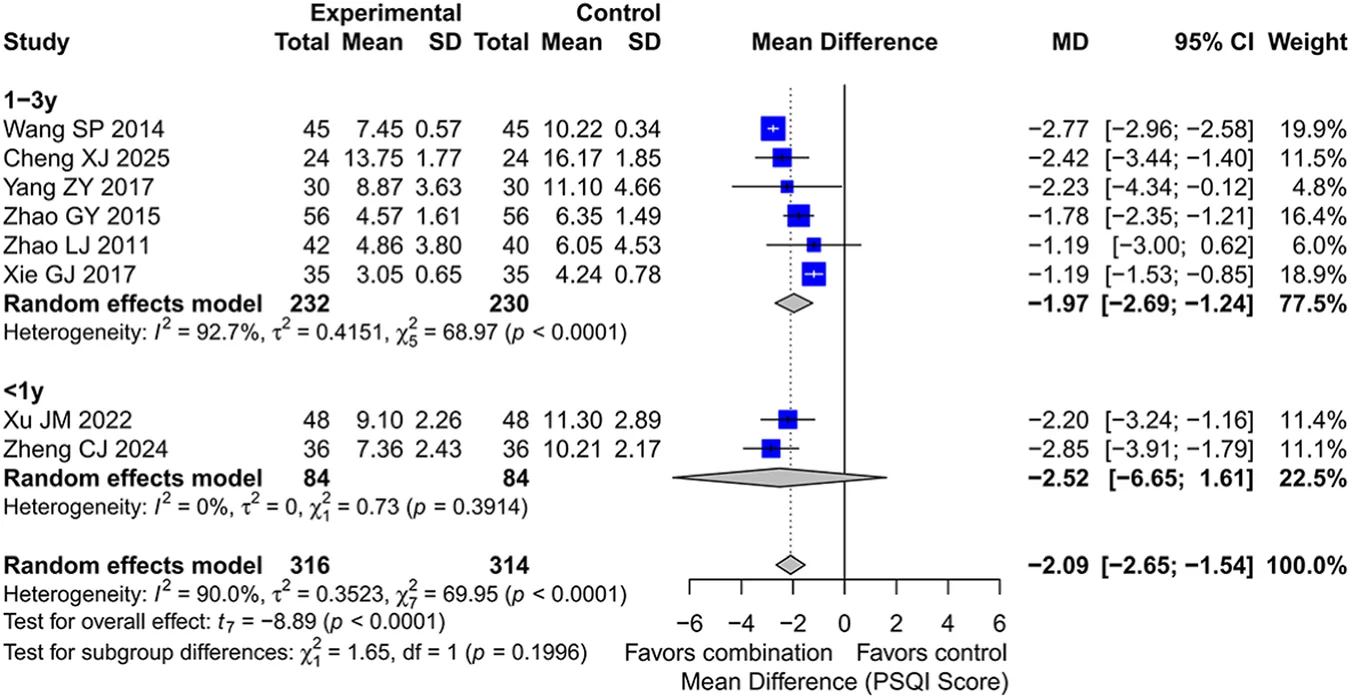
Subgroup forest plot of PSQI scores stratified by disease duration.

**FIGURE 8:**
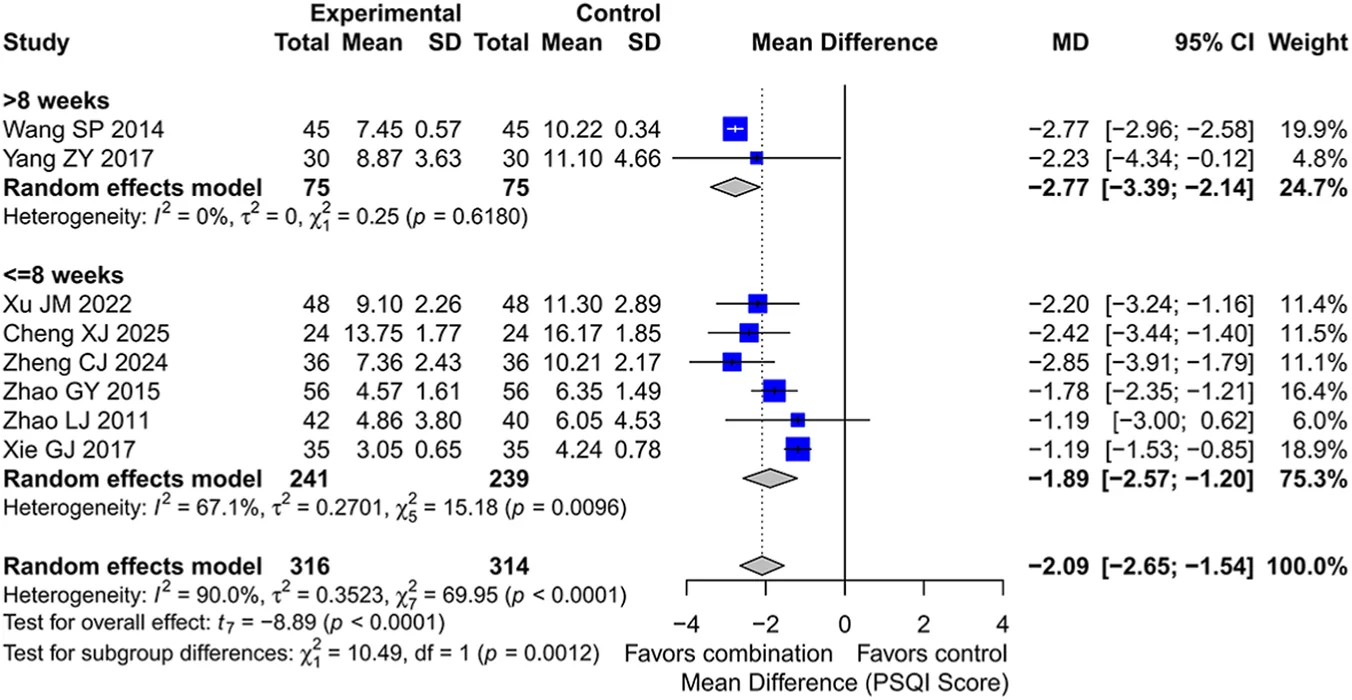
Subgroup forest plot of PSQI scores stratified by intervention duration.

### 3.6 Robustness and certainty of evidence

#### 3.6.1 Sensitivity analysis and publication bias

Leave-one-out sensitivity analyses verified the stability of pooled effect estimates for the total clinical effectiveness rate, PSQI, SDRS and adverse events (Figure 9; Supplementary Figure S6; Supplementary Figure S7; Supplementary Figure S8). Visual asymmetry was observed in funnel plots for the total clinical effectiveness rate outcome (Figure 10), and Egger’s regression test indicated significant publication bias for this endpoint (*P* = 0.0002) (Figure 11). In contrast, no statistically significant publication bias was detected for PSQI scores (*P* = 0.4848) and adverse events (*P* = 0.3958) (Supplementary Figure S9; Supplementary Figure S10). Notably, formal tests for publication bias possess low statistical power when fewer than 10 trials are included; accordingly, Egger-test results serve only as suggestive rather than definitive evidence. The trim-and-fill method was further applied to impute hypothetically missing unpublished studies. After adjustment, the pooled effect estimates for the three outcomes including total clinical effectiveness rate, PSQI, and adverse events remained statistically significant (adjusted *P* < 0.001), confirming the robustness of the main conclusions (Supplementary Figure S11; Supplementary Figure S12; Supplementary Figure S13). In addition, we performed a sensitivity analysis excluding the potential outlier study (Zhao et al., 2011), whose 95% confidence interval crossed zero and deviated from other included trials. After removal of this study, the pooled MD for PSQI changed slightly from −2.09 (95% CI −2.62 to −1.57, *I* ^*2*^ = 90.0%) to −2.15 (95% CI −2.70 to −1.60, *I* ^*2*^ = 91.2%). The direction and magnitude of the pooled effect remained largely consistent, suggesting that the primary finding of PSQI was not materially driven by this single extreme study. Nevertheless, substantial between-study heterogeneity persisted after outlier exclusion, indicating that other underlying factors contributed to the observed heterogeneity.

**FIGURE 9:**
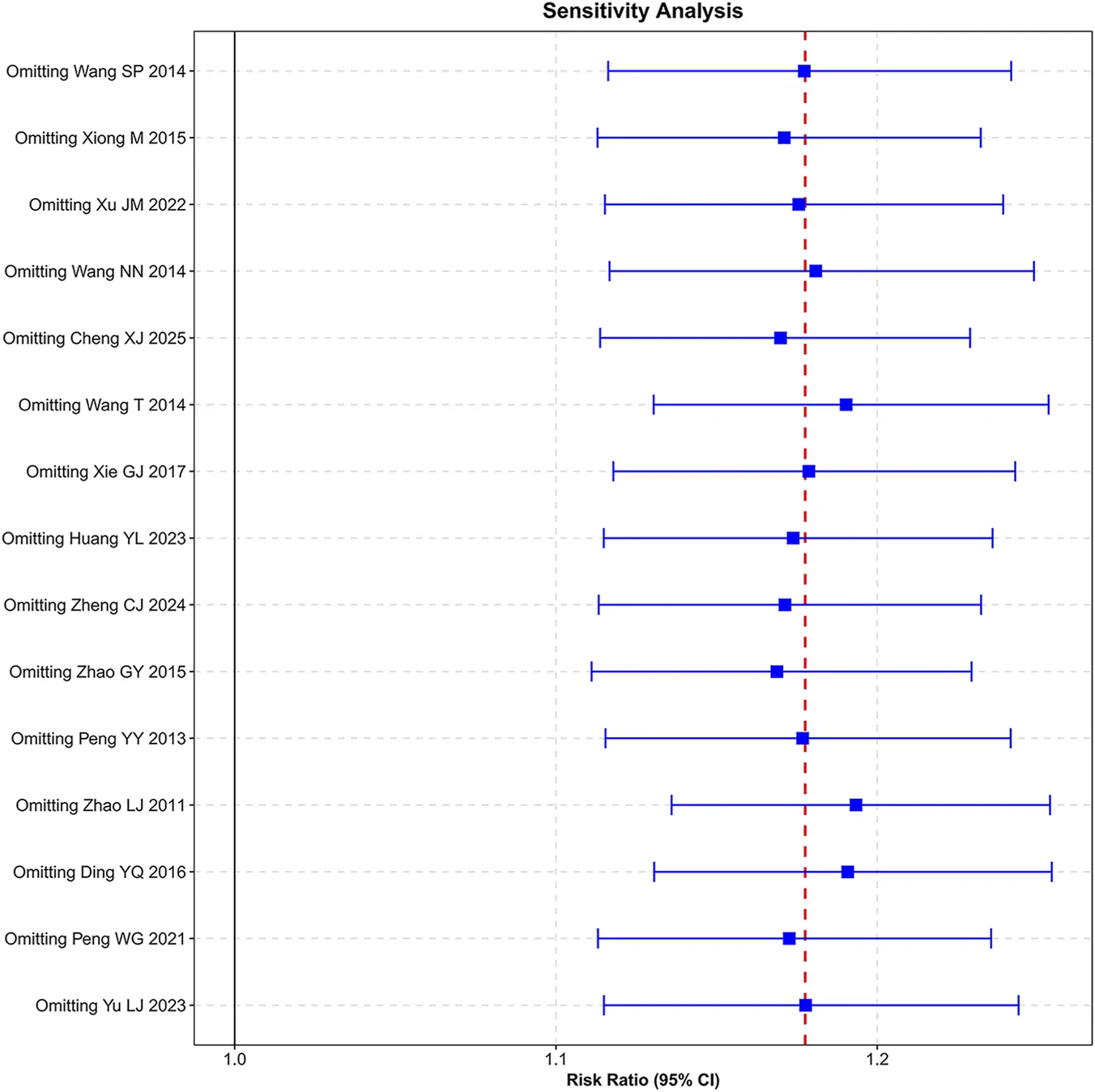
Leave-one-out sensitivity analysis plot for total clinical effectiveness rate.

**FIGURE 10:**
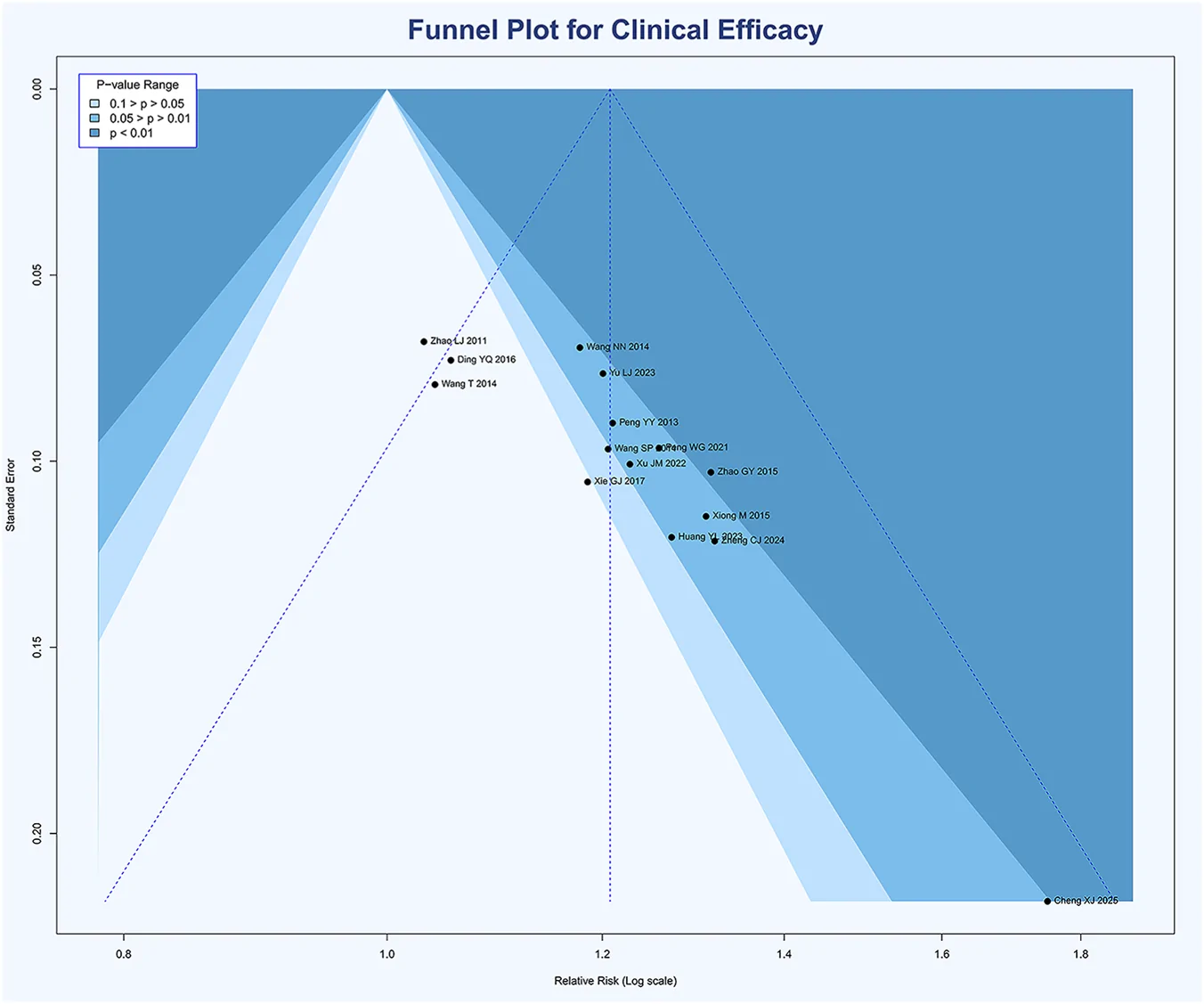
Funnel plot for publication bias assessment of total clinical effectiveness rate.

**FIGURE 11:**
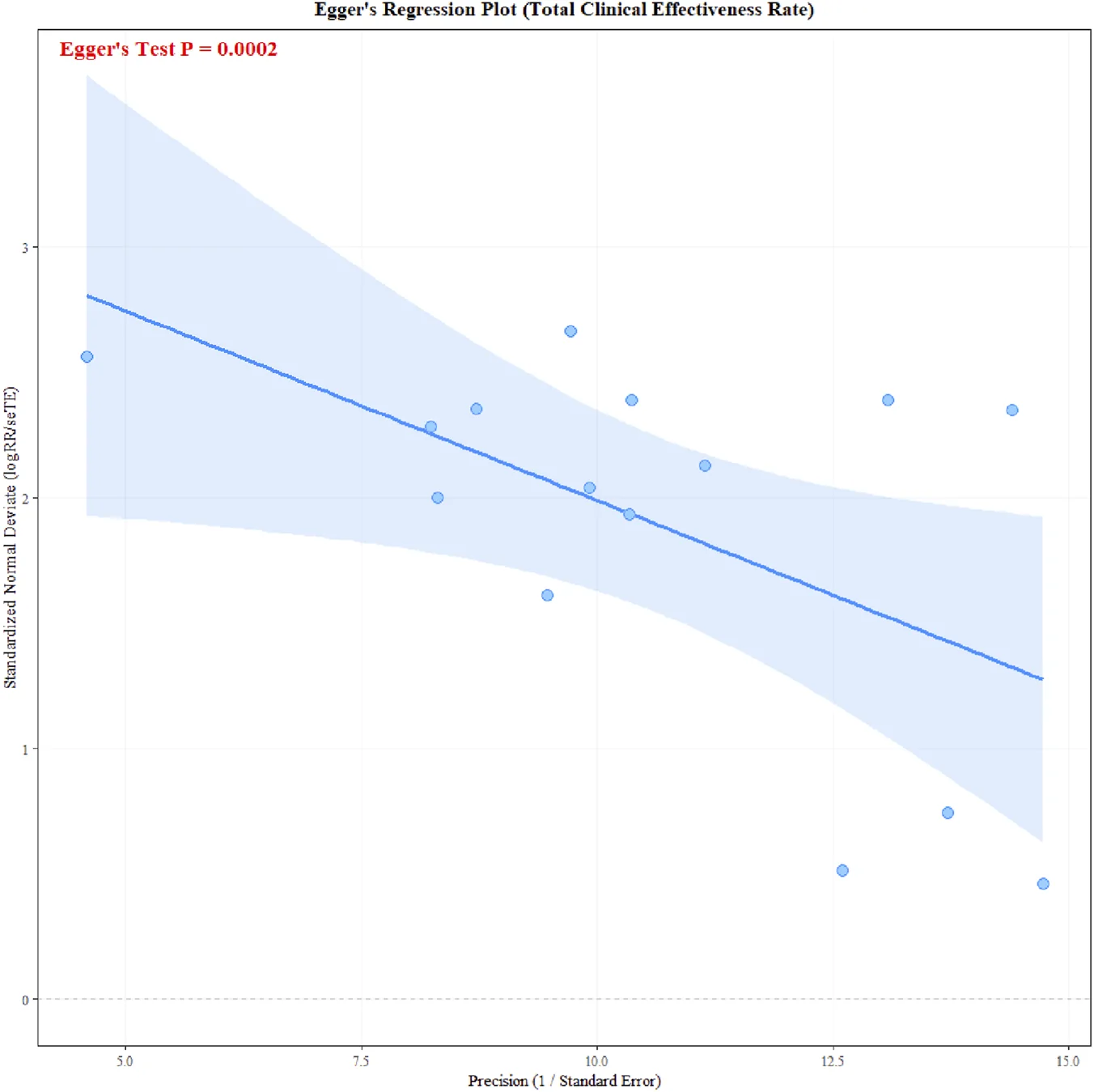
Egger’s regression plot for publication bias detection of total clinical effectiveness rate.

#### 3.6.2 P-curve analysis for evidential value

A critical divergence was observed in the evidential value of the efficacy outcomes:

Total Clinical Effectiveness Rate: The P-curve analysis indicated a lack of evidential value (Z = −0.61, *P* = 0.27; Power = 9%), suggesting that the significant meta-analytic result for this subjective outcome may be influenced by p-hacking or selective reporting (Figure 12).

**FIGURE 12:**
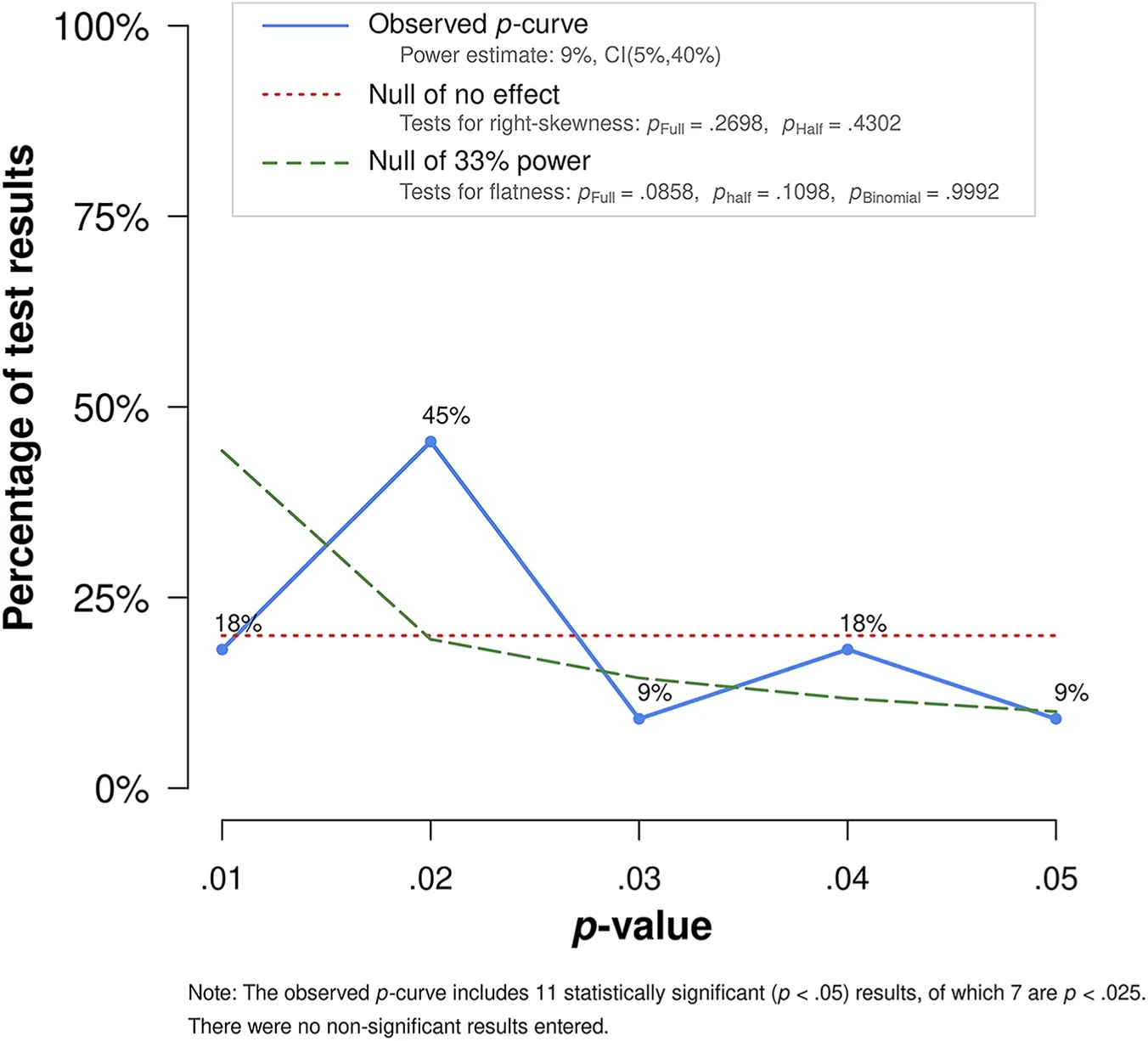
P-curve analysis evaluating evidential value for total clinical effectiveness rate.

PSQI Score: Conversely, the P-curve for PSQI demonstrated strong evidential value (Z = −11.48, *P <* 0.0001; Power = 99%), supporting the existence of genuine evidential value for improvements in PSQI-measured sleep quality (Figure 13).

**FIGURE 13:**
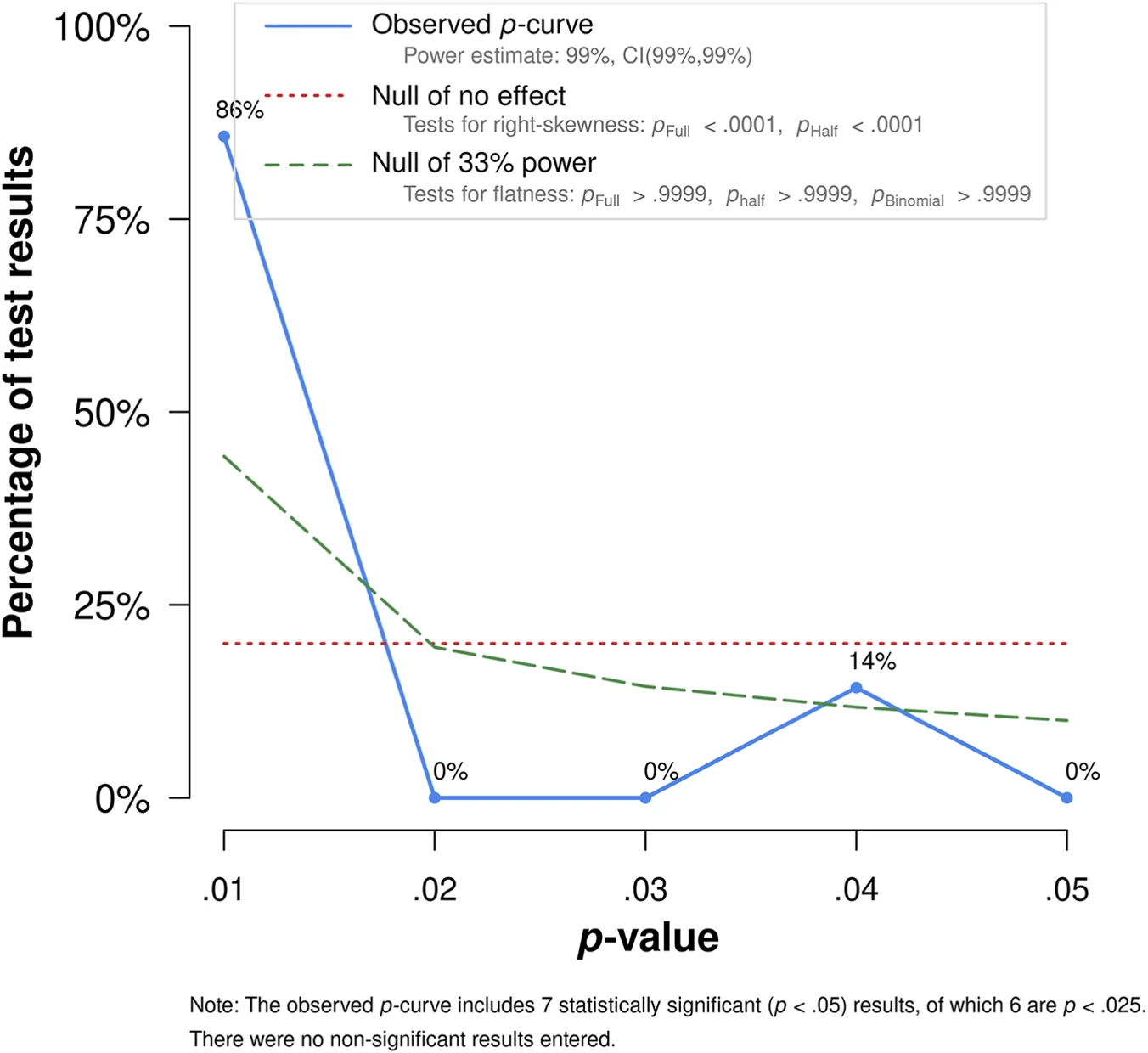
P-curve analysis evaluating evidential value for Pittsburgh Sleep Quality Index (PSQI).

#### 3.6.3 GRADE assessment

The certainty of evidence was appraised using the GRADE framework (Supplementary Figure S14): The certainty of evidence for each outcome was rated as “high”, “moderate”, “low”, or “very low” based on five domains: risk of bias, inconsistency, indirectness, imprecision, and publication bias. High-certainty evidence implies that the estimated effect is likely close to the true effect, whereas low or very low-certainty evidence suggests that the estimated effect may differ substantially from the true effect.

Regarding “risk of bias”, the evidence was downgraded because none of the included randomized controlled trials (RCTs) provided detailed descriptions of blinding. Regarding inconsistency, we assessed heterogeneity using the I^2^ statistic. The evidence for PSQI and SDRS scores was downgraded because the I^2^ values exceeded 50%, indicating significant heterogeneity.

Regarding “Imprecision”, we considered both sample size and the width of the 95% confidence intervals (CIs). The evidence for the SDRS score was downgraded due to wide confidence intervals.

We reported the results using standard GRADE narrative statements: high-certainty evidence is described with declarative statements; moderate-certainty with “probably”; low-certainty with “may”; and very low-certainty with “very uncertain.” When multiple measures existed for a single outcome, we compared their certainty ratings; if they differed, we prioritized reporting the measure with the higher certainty. If the ratings were identical, we reported the most commonly used measure.

## 4 Discussion

### 4.1 Summary of clinical efficacy

This systematic review and meta-analysis of 16 randomized controlled trials (RCTs) provides suggestive evidence supporting the integration of CCPPs with biomedicine pharmacotherapy for elderly patients with primary insomnia. Our findings suggest that this combined approach may yield superior clinical outcomes compared to biomedicine monotherapy, characterized by a significantly higher total clinical effectiveness rate (RR = 1.21) and a substantial reduction in PSQI scores (MD = −2.09). This therapeutic advantage is particularly critical given the rising prevalence of insomnia among the elderly population in China (Schiel and Spiegelhalder, 2020), where age-related physiological changes often render monotherapy insufficient or poorly tolerated. The data suggest that integrative medicine may overcome the “therapeutic ceiling” often encountered in geriatric sleep management.

### 4.2 Theoretical and biological mechanisms

The observed clinical benefits are underpinned by distinct yet complementary mechanisms of action. Primary insomnia in the elderly is biologically characterized by disruptions in neurotransmitter homeostasis, notably reduced serotonin (5-HT) levels and elevated pro-inflammatory cytokines such as TNF-α and IL-1β (Yu et al., 2021). While Western medications like benzodiazepines (BZDs) and non-benzodiazepines target GABA-ergic pathways to induce sleep, they do not address the underlying inflammatory or metabolic imbalances. From the perspective of Traditional Chinese Medicine, insomnia originates from yin-yang imbalance within the visceral systems of the heart, liver and kidney (Yin, 2025). Elderly patients with insomnia can be further differentiated into several TCM syndromes, including heart-liver blood deficiency, liver-yang ascendant generating internal wind, heart-kidney disharmony, liver-qi stagnation, heart-blood stasis, and yin-deficiency with fire effulgence. Corresponding therapeutic approaches, such as tonifying heart-blood, pacifying liver-yang to extinguish wind, harmonizing heart and kidney, soothing the liver to relieve constraint, activating blood circulation, and nourishing yin to clear heat, are applied for syndrome-targeted management.

The CCPPs included in this meta-analysis exert syndrome-specific therapeutic effects. Wuling Capsule, Tianwang Buxin Pill, Yishen Yangxin Tablets and Yimianda Tablets tonify the kidney and nourish the heart while calming the mind, and are mainly indicated for elderly patients with heart-kidney disharmony. Bailemian Capsule functions to nourish yin, clear heat and calm the spirit, which is suitable for patients with yin-deficiency-fire hyperactivity. Zaoren Anshen Liquid focuses on nourishing the heart and tranquilizing the mind for elderly patients with chronic insomnia. Shensong Yangxin Capsule, Qiye Anshen Tablets and Yindan Xingnaotong Capsule activate blood circulation to remove stasis for patients presenting with heart-blood stasis. Songling Xuemaikang Capsule pacifies liver-yang and calms the mind for elderly chronic insomnia attributed to liver-yang ascendant generating internal wind. For older adults suffering from long-term anxiety-related insomnia with liver-qi stagnation, Shumian Capsule can soothe liver constraint and tranquilize the heart-spirit. It should be noted that these mechanistic interpretations are derived from TCM theories and official drug instructions, and cannot be directly validated by the present meta-analysis.

### 4.3 Enhanced safety profile in the geriatric population

Beyond therapeutic benefits, our meta-analysis revealed a lower incidence of documented mild adverse reactions in participants receiving combined therapy versus biomedicine monotherapy. All adverse events extracted from primary trials were mild, transient and self-limiting, including nausea and dizziness. Four included studies (Peng, 2013; Zhao et al., 2011; Ding, 2016; Wang T. et al., 2014) reported that adjunctive CCPPs could improve clinical efficacy while lowering the dosage of biomedical hypnotics. Pooled estimates demonstrated that the relative risk of recorded adverse events was reduced by 53% in the combination arm compared with monotherapy (RR = 0.47). It is speculated that adjuvant CCPPs might allow lowered doses or shortened courses of biomedical hypnotics in clinical practice. Nevertheless, this observation is derived from a small subset of included trials and requires confirmation in larger prospective studies.

### 4.4 Exploration of heterogeneity

A key strength of this study is the granular exploration of heterogeneity, particularly regarding PSQI scores. Our subgroup analysis identified “disease duration” as a critical determinant of treatment response. Heterogeneity dropped dramatically (I^2^ = 0%) in patients with a disease duration of less than 1 year, suggesting that early intervention with integrative therapy yields more consistent and predictable outcomes. In contrast, treatment duration (>8 weeks vs. ≤8 weeks) and biomedicine class (BZDs vs. non-BZDs) did not significantly explain the variability. This implies that the chronicity of insomnia may lead to more complex neurobiological changes that are harder to reverse, highlighting the importance of initiating combined treatment in the early stages of the disorder. Furthermore, we conducted an exploratory subgroup analysis stratified by TCM syndrome pattern for PSQI. Given that primary trials rarely documented the TCM syndrome types of participants, grouping was implemented according to the main therapeutic indications of each CCPP. No statistically significant difference was detected across TCM-syndrome subgroups in the between-subgroup test, although varying effect sizes were observed among distinct syndrome subgroups. Notably, the stasis of heart blood subgroup showed no significant pooled effect and exhibited extremely high heterogeneity, whereas consistent benefits were observed in several other syndrome subgroups. Nevertheless, each syndrome subgroup included merely one or two RCTs. Multiple testing increases the risk of type I error, and statistical power is limited. This observation provides preliminary clinical clues for individualized syndrome-based treatment but requires verification in future studies that clearly report patients’ TCM differentiation and recruit more trials for each syndrome subtype.

### 4.5 Critical appraisal: P-curve and GRADE assessment

This study distinguishes itself from previous meta-analyses (Shi et al., 2021; Zhu et al., 2023) by employing rigorous methodological tools to assess evidence quality. A striking divergence was observed between the “total clinical effectiveness rate” and PSQI scores. While the total clinical effectiveness rate appeared statistically significant, P-curve analysis indicated it lacked evidential value (Power = 9%), suggesting it may be driven by p-hacking or publication bias. Conversely, the PSQI score demonstrated strong evidential value (Power = 99%), supporting the existence of genuine evidential value for improvements in PSQI-measured sleep quality. This finding is pivotal: it warns clinicians and researchers that the “total clinical effectiveness rate”-a subjective and non-standardized metric-is unreliable, and future trials should prioritize validated continuous measures like PSQI. Furthermore, the GRADE assessment rated the certainty of evidence as “Very Low” to “Moderate,” primarily due to the universal lack of blinding in the included studies. This underscores the urgent need for higher-quality RCTs to substantiate these promising findings.

### 4.6 Limitations

The results must be interpreted in light of several limitations. First, all included studies were conducted in China, which may limit generalizability to other ethnic populations due to genetic or cultural differences in TCM usage. Second, safety assessment was limited by poor adverse event recording across included RCTs. Although we summarized all reported mild adverse reactions in Table 2, no trials tracked key geriatric risks such as falls, cognitive impairment, sedation, dependence, withdrawal, or organ dysfunction. Selective underreporting may overestimate the safety advantage of combined therapy. Future trials should use standardized adverse event checklists and full laboratory testing to minimize reporting bias. In addition, none of the included studies evaluated drug-drug interactions between CCPPs botanical metabolites and biomedical hypnotics. Given the multicomponent nature of CCPPs and the physiological vulnerability of older adults, uncharacterized interaction risks remain possible. Third, risk of bias appraisal requires careful consideration. No included RCT implemented blinding of participants or assessors. As primary outcomes including total clinical effectiveness rate are subjective, absence of blinding introduces considerable potential for performance and detection bias. Furthermore, nearly all original trials lacked prospective registration, limiting our ability to verify pre-specified outcome plans and increasing the risk of selective outcome reporting. These methodological shortcomings contributed to downgrading evidence certainty in the GRADE assessment. Fourth, although we excluded severe comorbidities, the influence of undiagnosed subclinical conditions in this elderly patient cohort cannot be entirely ruled out. Finally, the “very low” GRADE rating for efficacy outcomes reflects the methodological flaws inherent in the primary literature. While blinding poses practical challenges for botanical-drug trials, this nevertheless constitutes a critical methodological shortcoming.

## 5 Conclusion and future directions

CCPPs combined with biomedicine may improve sleep quality and may reduce reported adverse events in elderly patients with primary insomnia. Although the pooled analyses demonstrated favorable outcomes for PSQI and adverse events, the overall evidence certainty is limited due to prevalent methodological limitations, substantial inter-study heterogeneity, and potential publication bias across the included trials. Notably, the total clinical effectiveness rate showed poor evidential value and may be unreliable owing to reporting bias, while validated PSQI outcomes presented robust evidential support. Clinicians are recommended to prioritize objective scale-based findings when interpreting relevant results. Further large-scale, multicenter, double-blind, placebo-controlled randomized controlled trials are required to confirm these preliminary findings and establish rigorous evidence for the clinical application of combined CCPPs and biomedicine therapy.

## Data Availability

The original contributions presented in the study are included in the article/Supplementary Material, further inquiries can be directed to the corresponding authors.

## Supplementary material

SUPPLEMENTARY FIGURE S1 Distribution of clinical efficacy of included studies between the combination group and control group.

SUPPLEMENTARY FIGURE S2 Composition of clinical efficacy stratified by individual included study.

SUPPLEMENTARY FIGURE S3 Forest plot of standardized mean difference for SDRS score.

SUPPLEMENTARY FIGURE S4 Subgroup forest plot of PSQI scores stratified by medicine type.

SUPPLEMENTARY FIGURE S5 Subgroup forest plot of PSQI scores stratified by TCM syndrome type.

SUPPLEMENTARY FIGURE S6 Sensitivity analysis of PSQI score.

SUPPLEMENTARY FIGURE S7 Sensitivity analysis of SDRS score.

SUPPLEMENTARY FIGURE S8 Sensitivity analysis of adverse events.

SUPPLEMENTARY FIGURE S9 Egger's regression plot for publication bias assessment of PSQI score.

SUPPLEMENTARY FIGURE S10 Egger's regression plot for publication bias assessment of adverse events.

SUPPLEMENTARY FIGURE S11 Egger's regression plot with trim-and-fill method to assess publication bias for total clinical effectiveness rate.

SUPPLEMENTARY FIGURE S12 Egger's regression plot with trim-and-fill method to assess publication bias for PSQI scores.

SUPPLEMENTARY FIGURE S13 Egger's regression plot with trim-and-fill method to assess publication bias for adverse events.

SUPPLEMENTARY FIGURE S14 GRADE quality assessment summary table.
