# Supplementary material for: Efficacy and safety of commercial Chinese polyherbal preparations combined with biomedicine for elderly patients with primary insomnia: a systematic review and meta-analysis of randomized controlled trials
Source: Front Pharmacol. 2026 Oct 23;17:1841014. doi: 10.3389/fphar.2026.1841014 (PMC13741095; doi:10.3389/fphar.2026.1841014)
Supplement: Supplementary file 1 [file Supplementaryfile1.pdf]

Appendix 1: Search Strategies for Seven Chinese-language Databases

Database	Search strategy
**CNKI:**	TKA=(失眠 + 不寐 + 睡眠障碍)*老年*(中药 + 中药复方 + 丸 + 颗粒 + 胶囊 + 片 + 中西医 + 联合)*(随机 + 随机对照)
**Wanfang**	全部:(失眠 OR 不寐 OR 睡眠障碍 OR 眠差 OR 睡眠困难) AND 全部:(老年) AND 全部:(中药 OR 中药复方 OR 颗粒 OR胶囊 OR 片 OR 丸 OR 中药 OR 联合 OR 中西医)AND 全部:(随机 OR 随机对照 OR RCT)
**VIP**	T=(失眠 OR 眠差 OR 入睡困难 OR 睡眠障碍) AND T=(老年) AND T=(中药 OR 中药复方 OR 丸 OR 颗粒 OR 胶囊 OR片 OR 中西医 OR 联合)AND M=(随机 OR 随机对照 OR rct)
**CBM**	#1 失眠[TI,AB] OR 不寐[TI,AB] OR 睡眠障碍[TI,AB] OR 失眠症[TI,AB]<br>#2 老年[TI,AB] OR 老年人[TI,AB] OR 老年性[TI,AB]<br>#3 中药[TI,AB] OR 中药复方[TI,AB] OR 丸[TI,AB] OR 颗粒[TI,AB] OR 胶囊[TI,AB] OR 片剂[TI,AB] OR 中西医结合[TI,AB] OR 联合治疗[TI,AB] OR 中成药[TI,AB]<br>#4 随机[TI,AB] OR 随机对照[TI,AB] OR 随机对照试验[TI,AB] OR RCT[TI,AB]<br>#5 #1 AND #2 AND #3 AND #4
**Pubmed**	("Elderly"[tiab]) AND ("Sleep Initiation and Maintenance Disorders"[MeSH Terms] OR insomnia[tiab] OR sleep disorder[tiab] OR sleep disturbance[tiab]) AND ("Drugs, Chinese Herbal"[MeSH Terms] OR Chinese herbal medicine[tiab] OR Chinese herbal formula[tiab] OR traditional Chinese medicine[tiab] OR Chinese patent medicine[tiab]) AND ("Randomized Controlled Trial"[Publication Type] OR randomized controlled trial[tiab] OR RCT[tiab])
**Cochrane**	#1 MeSH descriptor: [Sleep Initiation and Maintenance Disorders] explode all trees #2 (Sleep Wake Disorders):ti,ab,kw OR (Dyssomnias):ti,ab,kw OR (Sleep Disorders, Intrinsic):ti,ab,kw OR (Disorders of Excessive Somnolence):ti,ab,kw #3 #1 OR #2 #4 (elderly):ti,ab,kw OR (elder adult):ti,ab,kw OR (old adult):ti,ab,kw #5 #3 AND #4 #6 (Chinese patent medicine):ti,ab,kw OR (traditional Chinese medicine):ti,ab,kw OR (proprietary Chinese medicine):ti,ab,kw OR (integrated Chinese and Western medicine):ti,ab,kw OR (Integrated):ti,ab,kw OR (herbal):ti,ab,kw OR (chines traditional medicine):ti,ab,kw #7 #5 AND #6 #8 (aged):ti,ab,kw #9 #7 OR #8

**Embase**	(aged/exp OR elderly:ti,ab) AND (sleep initiation and maintenance disorder/exp OR insomnia:ti,ab OR "sleep disorder":ti,ab OR "sleep disturbance":ti,ab) AND ("Chinese herbal drug"/exp OR "Chinese herbal medicine":ti,ab OR "Chinese herbal formula":ti,ab OR "traditional Chinese medicine":ti,ab OR "Chinese patent medicine":ti,ab) AND ("randomized controlled trial"/exp OR "randomized controlled trial":ti,ab OR "randomized controlled trial":ti,ab OR RCT:ti,ab)
