# Supplementary material for: Efficacy and safety of commercial Chinese polyherbal preparations combined with biomedicine for elderly patients with primary insomnia: a systematic review and meta-analysis of randomized controlled trials
Source: Front Pharmacol. 2026 Oct 23;17:1841014. doi: 10.3389/fphar.2026.1841014 (PMC13741095; doi:10.3389/fphar.2026.1841014)
Supplement: Supplementary file 2 [file Supplementaryfile2.pdf]

Appendix 2: Taxonomic and Product Information of Included Commercial Chinese Polyherbal Preparations

General note for Appendix 2 Full formulation, ingredient composition and taxonomic information for each Commercial Chinese Polyherbal Preparation (CCPP) were retrieved from official NMPA- approved package inserts. Botanical nomenclature was cross- validated against Plants of the World Online (POWO). Animal- , mineral- and fossil- derived materia medica were referenced to the *Chinese Pharmacopoeia (2020 edition)*, as these taxa are not covered by POWO. The “Notes on reporting in primary RCTs” column documents what information was actually reported in the original RCT publications, which frequently only state the commercial CCPP product name without disclosing underlying formulation or botanical details.

CCPPs English name	Chinese name	Botanical drug components (validated taxonomy)	Dosage form	Source of information	Notes on reporting in primary RCTs
Shensong Yangxin Capsule	参松养心胶囊	*Panax ginseng* C.A.Mey. [Araliaceae] (root); *Ophiopogon japonicus* (L.f.) Ker-Gawl. [Asparagaceae] (root-tuber); *Cornus oficinalis* Siebold & Zucc. [Cornaceae] (fruit); *Salvia miltiorrhiza* Bunge [Lamiaceae] (root-rhizome); *Ziziphus jujuba* Mill. var. *spinosa* (Bunge) Hu ex H.F.Chow [Rhamnaceae] (seed, stir-fried); *Taxillus chinensis* (DC.) Danser [Loranthaceae] (branch-leaf); *Paeonia lactiflora* Pall. [Paeoniaceae] (root); Eupolyphaga Steleophaga (insect Polyphagidae); *Nardostachys chinensis* Batalin [Caprifoliaceae] (rhizome-root); *Coptis chinensis* Franch. [Ranunculaceae] (rhizome); *Schisandra sphenanthera* Rehd. & Wils. [Schisandraceae] (fruit); Os Draconis (fossil bone); Taxonomy reference for animal and fossil medicines: Chinese Pharmacopoeia 2020 (not available in POWO)	Capsule	Full botanical-drug composition was obtained from the NMPA-approved package insert; botanical nomenclature was validated against *Plants of the World Online (POWO)* and Chinese Pharmacopoeia (2020 edition).	Relevant RCTs only mentioned partial herbal ingredient names without full formulation details or formal taxonomic information. Comprehensive ingredient and taxonomic data were retrieved from official NMPA- approved package inserts.
Qiye Anshen Tablet	七叶安神片	*Panax notoginseng* (Burkill) F.H.Chen ex C.H.Chow [Araliaceae], leaf total saponins	Tablet	Full botanical-drug composition was obtained from the NMPA-approved package insert; botanical nomenclature was validated against *Plants of*	Relevant RCTs only mentioned partial herbal ingredient names without full formulation details or formal taxonomic information. Comprehensive ingredient and taxonomic data were retrieved from

				the World Online (POWO) and Chinese Pharmacopoeia (2020 edition).	official NMPA- approved package inserts.
Zaoren Anshen Oral Liquid	枣仁安神液	*Ziziphus jujuba* Mill. var. *spinosa* (Bunge) Hu ex H.F.Chow [Rhamnaceae] (seed, stir-fried); *Salvia miltiorrhiza* Bunge [Lamiaceae] (root-rhizome); *Schisandra chinensis* (Turcz.) Baill. [Schisandraceae] (fruit, vinegar-processed)	Oral Liquid	Full botanical-drug composition was obtained from the NMPA-approved package insert; botanical nomenclature was validated against *Plants of the World Online (POWO)* and Chinese Pharmacopoeia (2020 edition).	Relevant RCTs only mentioned partial herbal ingredient names without full formulation details or formal taxonomic information. Comprehensive ingredient and taxonomic data were retrieved from official NMPA- approved package inserts.
Zaoren Anshen Granules	枣仁安神颗粒	*Ziziphus jujuba* Mill. var. *spinosa* (Bunge) Hu ex H.F.Chow [Rhamnaceae] (seed, stir-fried); *Salvia miltiorrhiza* Bunge [Lamiaceae] (root-rhizome); *Schisandra chinensis* (Turcz.) Baill. [Schisandraceae] (fruit, vinegar-processed)	Granules	Full botanical-drug composition was obtained from the NMPA-approved package insert; botanical nomenclature was validated against *Plants of the World Online (POWO)* and Chinese Pharmacopoeia (2020 edition).	Relevant RCTs only mentioned partial herbal ingredient names without full formulation details or formal taxonomic information. Comprehensive ingredient and taxonomic data were retrieved from official NMPA- approved package inserts.
Yindan Xinnaotong Soft Capsule	银丹心脑通软胶 囊	*Ginkgo biloba* L. [Ginkgoaceae] (leaf); *Salvia miltiorrhiza* Bunge [Lamiaceae] (root-rhizome); *Erigeron breviscapus* (Vaniot) Hand.-Mazz. [Asteraceae] (whole-herb); *Gynostemma pentaphyllum* (Thunb.) Makino [Cucurbitaceae] (aerial parts); *Crataegus pinnatifida* Bunge [Rosaceae] (fruit); *Allium sativum* L. [Amaryllidaceae] (bulb); *Panax notoginseng* (Burkill) F.H.Chen ex C.H.Chow [Araliaceae] (root-rhizome); Blumea balsamifera (L.) DC. extract (Ai-pian); Taxonomy reference for	Capsule	Full botanical-drug composition was obtained from the NMPA-approved package insert; botanical nomenclature was validated against *Plants of the World Online (POWO)* and Chinese	Relevant RCTs only mentioned partial herbal ingredient names without full formulation details or formal taxonomic information. Comprehensive ingredient and taxonomic data were retrieved from official NMPA- approved package inserts.

		plant extract: Chinese Pharmacopoeia 2020 (not available in POWO)		Pharmacopoeia (2020 edition).	
Yishen Yangxin Anshen Tablet	益肾养心安神片	*Ziziphusjujuba* Mill. var. *spinosa* (Bunge) Hu ex H.F.Chow [Rhamnaceae] (seed, stir-fried); *Fallopia multiflora* (Thunb.) Haraldson [Polygonaceae] (root-rhizome, processed); *Morus alba* L. [Moraceae] (fruit); *Lilium brownii* F.E.Brown var. *viridulum* Baker [Liliaceae] (bulb); *Salvia miltiorrhiza* Bunge [Lamiaceae] (root-rhizome); *Ganoderma lucidum* (Curtis) P.Karst. [Polyporaceae] (fruiting-body); *Poria cocos* (Schw.) Wolf [Polyporaceae] (sclerotium); *Anemarrhena asphodeloides* Bunge [Asparagaceae] (rhizome); *Albiziajulibrissin* Durazz. [Fabaceae] (flower); *Chrysanthemum morifolium* Ramat. [Asteraceae] (flower)	Tablet	Full botanical-drug composition was obtained from the NMPA-approved package insert; botanical nomenclature was validated against *Plants of the World Online (POWO)* and Chinese Pharmacopoeia (2020 edition).	Relevant RCTs only mentioned partial herbal ingredient names without full formulation details or formal taxonomic information. Comprehensive ingredient and taxonomic data were retrieved from official NMPA- approved package inserts.
Tianwang Buxin Pellet	天王补心丹	*Rehmannia glutinosa* (Gaertn.) DC. [Orobanchaceae] (root); *Angelica sinensis* (Oliv.) Diels [Apiaceae] (root); *Asparagus cochinchinensis* (Lour.) Merr. [Asparagaceae] (root-tuber); *Ophiopogonjaponicus* (L.f.) Ker-Gawl. [Asparagaceae] (root-tuber); *Scrophularia ningpoensis* Hemsl. [Scrophulariaceae] (root-rhizome); *Platycodon grandiflorus* (Jacq.) A.DC. [Campanulaceae] (root); *Poria cocos* (Schw.)	Pellet	Full botanical-drug composition was obtained from the NMPA-approved package insert; botanical nomenclature was validated against *Plants of the World Online*	Relevant RCTs only mentioned partial herbal ingredient names without full formulation details or formal taxonomic information. Comprehensive ingredient and taxonomic data were retrieved from official NMPA- approved package

		Wolf [Polyporaceae] (sclerotium); *Polygala tenuifolia* Willd. [Polygalaceae] (root); *Ziziphus jujuba* Mill. var. *spinosa* (Bunge) Hu ex H.F.Chow [Rhamnaceae] (seed); *Platycladus orientalis* (L.) Franco [Cupressaceae] (seed); *Schisandra chinensis* (Turcz.) Baill. [Schisandraceae] (fruit); *Salvia miltiorrhiza* Bunge [Lamiaceae] (root-rhizome); *Codonopsis pilosula* (Franch.) Nannf. [Campanulaceae] (root); Cinnabar (mineral, HgS); Taxonomy reference for mineral medicine: Chinese Pharmacopoeia 2020 (not available in POWO)		*(POWO)* and Chinese Pharmacopoeia (2020 edition).	inserts.
Wuling Capsule	乌灵胶囊	*Xylaria nigripes* (Kl.) Sacc. [Xylariaceae], fermented mycelium powder (Wuling fungus powder); Taxonomy reference: GBIF and mycological literature (not available in POWO)	Capsule	Full botanical-drug composition was obtained from the NMPA-approved package insert; botanical nomenclature was validated against *Plants of the World Online (POWO)* and Chinese Pharmacopoeia (2020 edition).	Relevant RCTs only mentioned partial herbal ingredient names without full formulation details or formal taxonomic information. Comprehensive ingredient and taxonomic data were retrieved from official NMPA- approved package inserts.
Songling Xuemaikang Capsule	松龄血	*Pinus massoniana* Lamb. [Pinaceae] (fresh leaf-needle);	Capsule	Full botanical-drug	Relevant RCTs only mentioned partial

	脉康胶囊	*Pueraria lobata* (Willd.) Ohwi [Fabaceae] (root); Powder of nacre (animal shell layer); Taxonomy reference for nacre: Chinese Pharmacopoeia 2020 (not available in POWO)		composition was obtained from the NMPA-approved package insert; botanical nomenclature was validated against *Plants of the World Online (POWO)* and Chinese Pharmacopoeia (2020 edition).	herbal ingredient names without full formulation details or formal taxonomic information. Comprehensive ingredient and taxonomic data were retrieved from official NMPA- approved package inserts.
Shumian Capsule	舒眠胶囊	*Bupleurum chinense* DC. [Apiaceae] (root, wine-processed); *Ziziphusjujuba* Mill. var. *spinosa* (Bunge) Hu ex H.F.Chow [Rhamnaceae] (seed, stir-fried); *Albiziajulibrissin* Durazz. [Fabaceae] (bark); *Albiziajulibrissin* Durazz. [Fabaceae] (flower); *Paeonia lactiflora* Pall. [Paeoniaceae] (root, stir-fried); *Juncus efusus* L. [Juncaceae] (pith); Bombyx Batryticatus (dried infected larva); Cicadae Periostracum (insect exuvia); Taxonomy reference for animal medicines: Chinese Pharmacopoeia 2020 (not available in POWO)	Capsule	Full botanical-drug composition was obtained from the NMPA-approved package insert; botanical nomenclature was validated against *Plants of the World Online (POWO)* and Chinese Pharmacopoeia (2020 edition).	Relevant RCTs only mentioned partial herbal ingredient names without full formulation details or formal taxonomic information. Comprehensive ingredient and taxonomic data were retrieved from official NMPA- approved package inserts.
Bailemian Capsule	百乐眠胶囊	*Lilium brownii* F.E.Br. ex Miellez [Liliaceae] (bulb); *Eleutherococcus senticosus* (Rupr. & Maxim.) Maxim. [Araliaceae] (root-rhizome); *Fallopia multiflora* (Thunb.) Haraldson [Polygonaceae] (stem); *Albiziajulibrissin* Durazz. [Fabaceae] (flower); Concha Margaritifera Usta (animal shell); Gypsum Fibrosum (mineral); *Ziziphusjujuba* Mill. var. *spinosa* (Bunge) Hu ex H.F.Chow [Rhamnaceae] (seed); *Poria cocos* (Schw.) Wolf [Polyporaceae] (sclerotium); *Polygala tenuifolia* Willd. [Polygalaceae] (root); *Scrophularia ningpoensis* Hemsl. [Scrophulariaceae] (root-rhizome); *Rehmannia glutinosa*	Capsule	Full botanical-drug composition was obtained from the NMPA-approved package insert; botanical nomenclature was validated against *Plants of the World Online (POWO)* and Chinese Pharmacopoeia (2020 edition).	Two RCTs used this preparation; one trial reported herbal constituent information, while the other only stated the commercial preparation name without listing herbal components.

		(Gaertn.) DC. [Orobanchaceae] (root); *Ophiopogonjaponicus* (L.f.) Ker-Gawl. [Asparagaceae] (root-tuber); *Schisandra chinensis* (Turcz.) Baill. [Schisandraceae] (fruit); *Juncus efusus* L. [Juncaceae] (pith); *Salvia miltiorrhiza* Bunge [Lamiaceae] (root-rhizome); Taxonomy reference for animal and mineral medicines: Chinese Pharmacopoeia 2020 (not available in POWO)			
Yimianda Tablet	益眠达片	*Albiziajulibrissin* Durazz. [Fabaceae] (bark); *Fallopia multiflora* (Thunb.) Haraldson [Polygonaceae] (stem); *Rehmannia glutinosa* (Gaertn.) DC. [Orobanchaceae] (root, raw); *Rehmannia glutinosa* (Gaertn.) DC. [Orobanchaceae] (root, processed); *Schisandra chinensis* (Turcz.) Baill. [Schisandraceae] (fruit); *Patrinia scabiosaefolia* Fisch. ex Trevir. [Caprifoliaceae] (herb); *Ziziphusjujuba* Mill. [Rhamnaceae] (fruit);	Tablet	Full botanical-drug composition was obtained from the NMPA-approved package insert; botanical nomenclature was validated against *Plants of the World Online (POWO)* and Chinese Pharmacopoeia (2020 edition).	Relevant RCTs only mentioned partial herbal ingredient names without full formulation details or formal taxonomic information. Comprehensive ingredient and taxonomic data were retrieved from official NMPA- approved package inserts.
